# Preparation and Pharmacokinetics of Brain-Targeted Nanoliposome Loaded with Rutin

**DOI:** 10.3390/ijms252111404

**Published:** 2024-10-23

**Authors:** Changxu Wu, Jinwu Zhang, Shisen Yang, Chunzi Peng, Maojie Lv, Jing Liang, Xiaoning Li, Liji Xie, Yingyi Wei, Hailan Chen, Jiakang He, Tingjun Hu, Zhixun Xie, Meiling Yu

**Affiliations:** 1 Guangxi Key Laboratory of Animal Breeding, Disease Control and Prevention, College of Animal Science and Technology, Guangxi University, Nanning 530004, China; 13054921763@163.com (C.W.);; 2 Guangxi Key Laboratory of Veterinary Biotechnology, Guangxi Veterinary Research Institute, Nanning 530001, Chinaxiezhixun@126.com (Z.X.)

**Keywords:** nanoliposome, brain-targeted Rutin, blood–brain barrier, pharmacokinetics

## Abstract

Rutin is a flavonoid compound with potential for treating Alzheimer’s disease, preventing brain damage, mitigating cerebral ischemia–reperfusion injury, and exhibiting anti-glioblastoma activity. However, its efficacy is limited by its low solubility, poor bioavailability, and limited permeability across the blood–brain barrier (BBB). To enhance the bioavailability and brain-targeting ability of Rutin, transferrin-modified Rutin liposome (Tf-Rutin-Lip) was developed using liposomes as a delivery system. Rutin liposomes were prepared using the thin-film dispersion method, and the preparation conditions were optimized using the response surface methodology. Then, transferrin (Tf) was incorporated into the liposomes through covalent modification, yielding Tf-Rutin liposomes. The toxicity of these liposomes on bEnd.3 cells, as well as their impact on the tight junctions of these cells, was rigorously evaluated. Additionally, in vitro and in vivo experiments were conducted to validate the brain-targeting efficacy of the Tf-Rutin liposomes. A susceptible detection method was developed to characterize the pharmacokinetics of Tf-Rutin-Lip further. The optimized conditions for the preparation of Tf-Rutin-Lip were determined as follows: a lipid-to-cholesterol ratio of 4.63:1, a drug-to-lipid ratio of 1:45.84, a preparation temperature of 42.7 °C, a hydration volume of 20 mL, a sonication time of 10 min, a surfactant concentration of 80 mg/mL, a DSPE-MPEG-2000 concentration of 5%, and a DSPE-PEG2000-COOH to DSPE-MPEG-2000 molar ratio of 10%. The liposomes did not affect the cell activity of bEnd.3 cells at 24 h and did not disrupt the tight junction of the blood–brain barrier. Tf-modified liposomes were taken up by bEnd.3 cells, which, in turn, passed through the BBB, thus improving liposomal brain targeting. Furthermore, the results of pharmacokinetic experiments showed that the C_max_, AUC_0-∞_, AUC_0-t_, MRT_0-∞_, and t_1/2_ of Tf-Rutin-Lip increased 1.99-fold, 2.77-fold, 2.58-fold, 1.26-fold, and 1.19-fold compared to those of free Rutin solution, respectively. These findings suggest that Tf-Rutin-Lip is brain-targeted and may enhance the efficacy of Rutin in the treatment of brain disorders.

## 1. Introduction

Brain disorders, such as brain cancer and the impaired functional integrity of the central nervous system (CNS), are among the most prevalent, devastating, and therapeutically challenging pathologies [1]. Despite diligent efforts made by the scientific community to devise effective treatment strategies for pathologies associated with dysfunctional brain function, ranging from diverse brain tumor types to neuroinflammatory conditions and neurodegenerative disorders, the current therapeutic modalities exhibit limited efficacy, often failing to halt disease progression [1].

**Figure 1:**
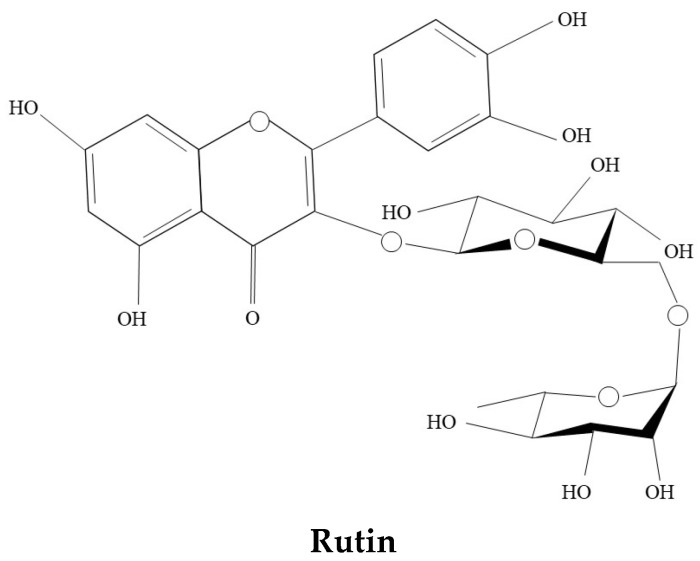
Chemical structure of Rutin.

Rutin, known as quercetin-3-*O*-rutinoside and vitamin P, is a lipophilic constituent that demonstrates solubility in organic solvents such as pyridine, methanol, and ethanol [2,3]. Chemically, Rutin (Figure 1) is a flavonol-type polyphenol consisting of the flavonol quercetin and the disaccharide rutinose, which exhibit diverse biological activities, including anti-inflammatory [4], antioxidant [5], hypoglycemic [6], antitumor [7,8], antiviral [9], and neuroprotective effects [10]. It has been reported that Rutin formulations remarkably attenuate the activation of neuroinflammation in both the peripheral and central nervous systems in zebrafish via regulating the expression of nuclear factor kappa-B (NF-κB) pathway-related genes, inhibiting NF-κB activation, and reducing interleukin (IL)-1β, IL-6, and tumor necrosis factor (TNF)-α production in C6 lineage cells [8,11]. Rutin supplementation regulates the expression of cyclic AMP response element binding protein (CREB) and brain-derived neurotrophic factor (BDNF), and alleviates colistin-induced inflammation, oxidative stress, apoptosis, as well as histopathological and immunohistochemical alterations [8]. It further promotes neuronal growth and repair, thereby mitigating colistin-induced neurotoxicity in male rats [8]. In addition, Rutin can bind to angiotensin-converting enzyme 2 (ACE2) for the treatment of reperfusion injury following acute ischemic stroke [12], and alleviate the reduction of neuroprotective factors in diabetic retina [13]. Furthermore, Rutin exerts antitumor effects in vivo and in vitro, showing cytotoxic effects, antiproliferative, antimigration, proapoptotic, and morphogenic effects on GL-15 or C6 glioblastomas (GBM) cells, reducing vascular endothelial growth factor secretion in U343 and U118 GBM cells and the formation of tumors in the brain of Wistar rats after 30 days of U-251 GBM cell xenotransplantation [7,8,14,15,16]. 

Though Rutin has been widely used in various therapeutic areas, its poor solubility, poor stability, inadequate absorption, and low bioavailability severely limit its clinical application. Additionally, the presence of the blood–brain barrier significantly hinders drug delivery, posing a major challenge in brain drug development and therapy [16]. To overcome the existing limitations in the development of highly effective drugs for the treatment of brain disorders, the targeted delivery of bioactive molecules using modern nanomedicine tools is promising [17,18,19]. The common nano-delivery systems include liposomes, nanoparticles, hydrogels, inclusion compounds, and nanoemulsions [20]. It is well-known that nanoparticles, hydrogels, and liposomes due to their excellent biocompatibility and encapsulation capabilities, are often used as efficient drug delivery carriers [20]. They can stably and effectively encapsulate rutin, enhancing its bioavailability and thus achieving more desirable therapeutic effects [21]. However, different delivery systems exhibit distinct characteristics. Nanoparticles, with their small size and high surface-to-volume ratio, can more easily enter cells. Hydrogels can maintain a specific shape and texture, thereby sustaining the concentration of Rutin in local tissues and enhancing therapeutic efficacy [22], while liposomes are considered powerful drug delivery systems primarily due to their structural versatility, biocompatibility, biodegradability, and non-toxic, non-immunogenic nature [23,24,25,26]. Memar et al. [27] employed solid lipid nanoparticles (SLNs) to deliver Rutin, demonstrating greater anticancer and antibacterial effects in vitro compared to pure Rutin. These effects were achieved by inducing apoptosis and generating reactive oxygen species (ROS) to inhibit the growth of HN5 cells [27].

However, the reticuloendothelial system (RES) can easily recognize and quickly eliminate conventional liposomes from plasma. Notably, PEGylated liposomes can significantly improve the drug’s circulation performance and increase drug accumulation in tumors to enhance antitumor efficacy [28]. Liposomes can also be targeted through modification with antibodies, nucleic acids (aptamers), peptides, whole proteins (e.g., transferrin, Tf), and small molecules, such as vitamins (e.g., folic acid) [29]. Tf, a widely employed ligand for drug targeting across the blood–brain barrier (BBB), efficiently facilitates drug accumulation in the brain [30]. By binding to the transferrin receptor (TfR) on the surface of the BBB, Tf is able to transport itself into the brain [30]. Transferrin-modified nanocarriers possess specific targeting capabilities for drugs [31]. For instance, Meihua Luo [31] prepared transferrin-modified porous silicon nanoparticles capable of crossing the BBB and delivering doxorubicin to glioblastoma. Transferrin (Tf)-modified poly(ethylene glycol)-phosphatidylethanolamine (mPEG-PE) micelles loaded with the poorly water-soluble drug R547, a potent and selective ATP-competitive cell-cycle protein-dependent kinase (CDK) inhibitor, exhibited high targeting efficiency and therapeutic efficacy against ovarian cancer in both in vitro and in vivo experiments [32].

**Figure 2:**
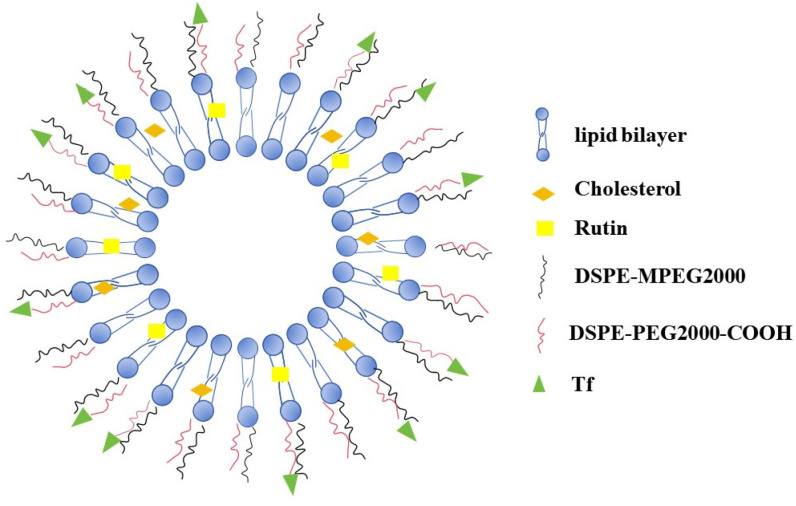
Structural diagram of a liposome.

In the current study, transferrin-modified liposomes encapsulating Rutin (Tf-Rutin-Lip) (Figure 2) were prepared via the film dispersion method. The liposomes were characterized by particle size, zeta potential, polydispersity index (PDI), encapsulation efficiency (EE), and in vitro drug release profiles. The brain-targeting capabilities of the liposomes were elevated in vitro and in vivo. Specifically, the in vivo pharmacokinetics of Tf-Rutin-Lip following a single oral administration in rats was determined using a sensitive and reliable high-performance liquid chromatography (HPLC) method. This study aims to innovatively utilize liposome technology to encapsulate Rutin and further modify it with transferrin (Tf), thereby targeting the delivery of Rutin to the brain, where the drug is difficult to penetrate. This original strategy will provide new theoretical support and practical avenues for treating brain disorders. Still, it may also open new ways to treat these difficult-to-conquer disorders. By precisely controlling the location and efficiency of Rutin, it is expected that its efficacy in treating brain disorders will be significantly improved, thus benefiting patients. This study provides a theoretical foundation for the further research and development of Rutin-based therapeutic formulations.

## 2. Results

### 2.1. Results of the Single-Factor Experiment

**Figure 3:**
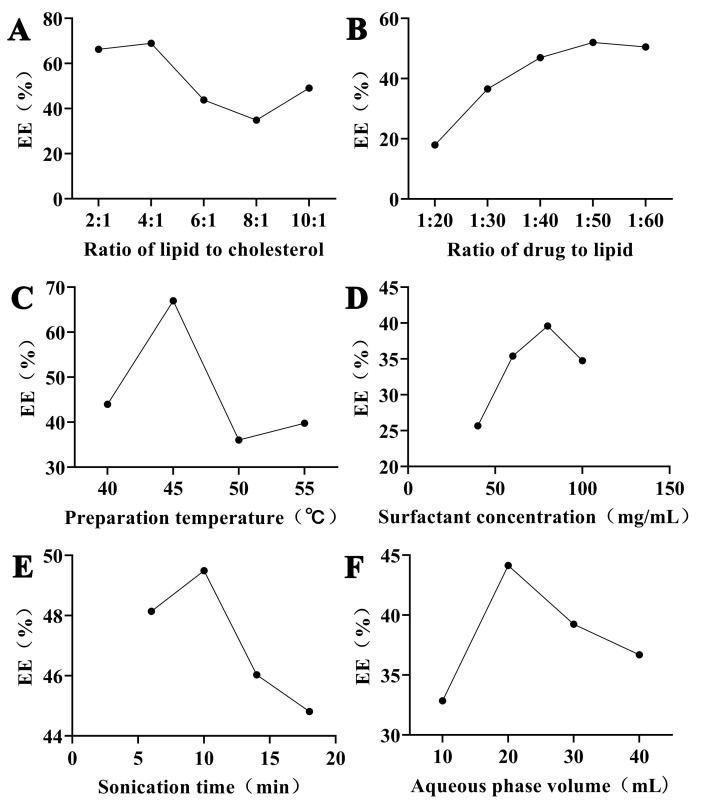
Effect of single-factors on the entrapment efficiency of Rutin liposomes. (**A**) Effect of soy lecithin-to-cholesterol ratio on the EE. (**B**) Effect of the rutin-to-soy lecithin ratio on the EE. (**C**) Effect of preparation temperature on the EE. (**D**) Effect of surfactant concentration (Tween-80) on the EE. (**E**) Effect of the sonication time on the EE. (**F**) Effect of saline volume on the EE.

According to Figure 3A, the maximum encapsulation efficiency (EE%) was achieved at a soy lecithin-to-cholesterol ratio of 4:1, with a maximum EE% of 68.96%, while the minimum EE% was observed at a ratio of 8:1. Consequently, soy lecithin-to-cholesterol ratio ranging from 2:1 to 6:1 was selected for further experiments. As shown in Figure 3B, EE% reached a maximum at a drug-to-soy lecithin ratio of 1:50 and then minimized at a ratio of 1:60. Therefore, the drug-to-lipid ratio range from 1:40 to 1:60 was employed in the following trials. As shown in Figure 3C, the preparation temperature had a profound effect on the EE; the maximum EE% of 66.99% was obtained at 45 °C. A preparation temperature between 40 °C and 50 °C was selected for further investigations. Aqueous phase volume, sonication time, and surfactant concentration had little effect on EE%. EE% reached a maximum of 39.59% at 80 mg/mL of surfactant (Figure 3D). The EE% gradually increased as the sonication time progressed from 5 to 10 min, achieving a maximum of 49.49% at 10 min (Figure 3E). As the hydration volume range from 10 mL to 40 mL, the EE% reached a maximum of 44.14% at 20 mL (Figure 3F).

### 2.2. Model Fitting and Statistical Analysis

The encapsulation efficiency (%, Y) of the nanoliposomes obtained from the experimental design using BBD are presented in Table 1. By applying multiple regression analysis to the experimental data, the relationship between the response variable (EE) and the independent variables was formulated using the following second-order polynomial equations:

Y = 69.92 + 2.40A + 0.7175B − 2.95C − 0.0675AB − 4.69AC + 5.84BC − 6.95A2 − 2.98B2 − 7.35C2 (R^2^ = 0.9787, *p* < 0.001)

An ANOVA was performed on the model, and the results showed that the model was significant (*p* < 0.05), while the lack of fit test of the model was not significant (*p* > 0.05), indicating that the model fit well and could sufficiently predict the optimal prescription (Table 2). The ANOVA result of the quadratic polynomial model for the entrapment efficiency of the Rutin nanoliposomes showed that the quadric term of the phospholipid-to-cholesterol ratio (A^2^) and the quadratic term of preparation temperature (C^2^) with *p* ≤ 0.0001 had the greatest effect on entrapment efficiency, followed by the interactive effect between the drug-to-lipid ratio and preparation temperature (BC), the interactive effect between the lipid-to-cholesterol ratio and preparation temperature (AC), the linear term of preparation temperature (C), the linear term of lipid-to-cholesterol ratio (A), and the quadratic term of drug-to-lipid ratio (B^2^), all with *p* < 0.01 (Table 1). Moreover, the linear term of lipid-to-cholesterol ratio (A) and the interactive effect between the lipid-to-cholesterol ratio and drug-to-lipid ratio (AB) had no significant effects (*p* > 0.05) on the nanoliposome EE%. The F value was calculated as 35.67, indicating that the model was significant. In addition, the calculated *p* value < 0.0001 confirmed that the model was suitable for the current experiment. Furthermore, the lack of fit (*p* = 0.789) was not significant, indicating that the pure errors were at their minimum.

### 2.3. Response Surface Analysis

**Figure 4:**
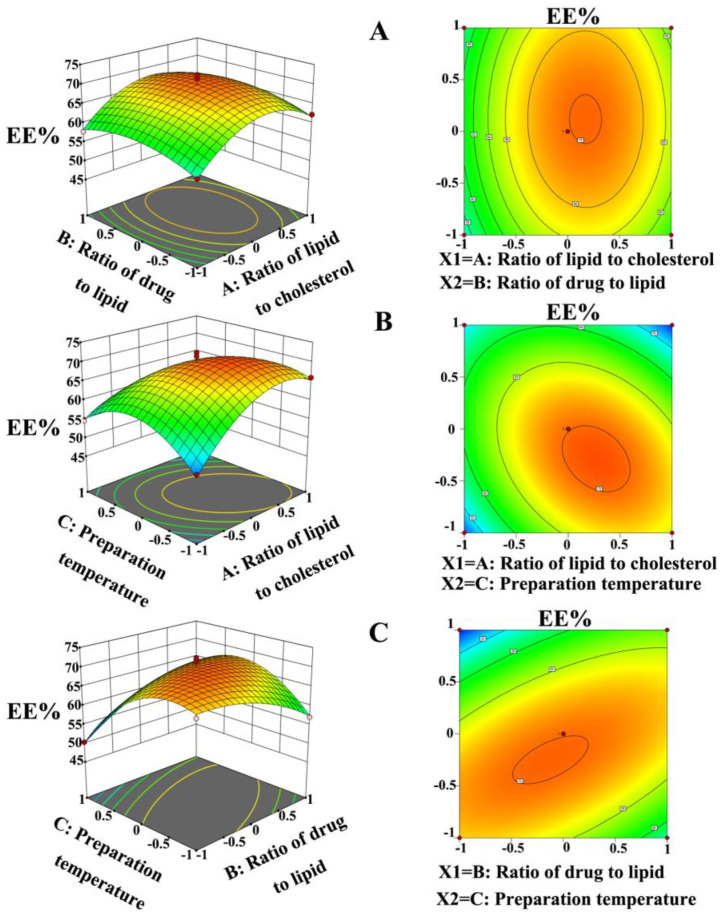
Three-dimensional response surface and two-dimensional contour plots showing the effects on entrapment efficiency of two variables (the other variable was kept at zero in the coded unit): interaction between (**A**) the lipid-to-cholesterol ratio and the drug-to-lipid ratio; (**B**) the lipid-to-cholesterol ratio and preparation temperature; (**C**) the drug-to-lipid ratio and preparation temperature.

Response surfaces were plotted using Design Expert to study the effects of independent variables and their interactions on the responses. Three-dimensional (3D) response surface plots and two-dimensional (2D) contour plots, as presented in Figure 3, illustrated the effects of two varieties on the response at a time point, where the others were kept at zero at the coded unit level. Maximum and minimum EE% values were obtained at 72.37 and 49.52%, respectively (Table 1). Response surface and contour plots (Figure 4A) showed the effect of the lipid-to-cholesterol ratio and drug-to-lipid ratio on the EE%, where the preparation temperature was fixed at 45 °C. The results showed that both the lipid-to-cholesterol ratio and the drug-to-lipid ratio had a secondary effect on the EE%, which began to increase and then decreased as the lipid-to-cholesterol ratio increased from 2:1 to 6:1. Consequently, there was an optimal value of the lipid-to-cholesterol ratio that corresponded to the highest EE%. Similarly, the EE% rapidly enhanced when the drug-to-lipid ratio increased from 1:40 to 1:50, reaching a plateau region with a maximum value, and then decreasing when the ratio increased from 1:50 to 1:60.

The relationship between the EE%, lipid-to-cholesterol ratio, and preparation temperature at a drug-to-lipid ratio of 1:50 was plotted and is shown in Figure 4B. A secondary effect of lipid-to-cholesterol ratio and preparation temperature on the EE% was observed. The effect of the lipid-to-cholesterol ratio on the EE% is shown in Figure 4A. By increasing the preparation temperature from 40 to 45 °C, the EE% increased from 52.31 to 72.37%, and then decreased to 49.52% by increasing the preparation temperature from 45 to 50 °C. Similarly, Figure 4C shows the response surface and contour plots, depicting the effects of the drug-to-lipid and preparation temperature on EE% with a fixed lipid-to-cholesterol ratio of 4:1. From the 3D plot, we concluded that both the drug-to-lipid ratio and preparation temperature displayed quadratic impacts on the response, which was consistent with the conclusions drawn from Figure 4A,B.

The combination of factor levels that achieved the optimum desirability according to the study aim for the lipid-to-cholesterol ratio, drug-to-lipid ratio, and preparation temperature were found to be 4.6:1 (*w*/*w*), 1:46 (*w*/*w*), and 42.7 °C, respectively. The optimized formulation produced under the above-mentioned optimum conditions was prepared, and the observed EE% values were found to be 70.81%. A verification experiment was performed under the optimized condition in order to validate the fitness of the equations. The optimized conditions were designed as follows: a lipid-to-cholesterol ratio of 4.6:1, a drug-to-lipid ratio of 1:46, and a preparation temperature of 42.7 °C. The mean experimental EE% value was 70.77%, which was close to the predicted values of 70.81% (Table 3). The results indicated that the RSM approach was adequate for optimizing the conditions for the preparation process.

### 2.4. Optimization Conditions for Formulating Tf-Rutin-Lip

The transferrin-modified Rutin liposomes were prepared using a post-insertion method on top of the normal liposomes. Single-factor optimization was performed for the molar ratio of total MPEG to phospholipids and the molar ratio of DSPE-PEG2000-COOH to DSPE-MPEG2000, both key factors.

**Figure 5:**
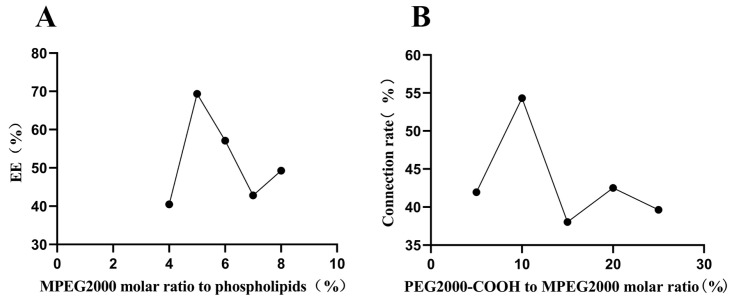
Single-factor optimization of Tf-Rutin-Lip. (**A**,**B**) indicate the effect of the molar ratio of MPEG to phospholipids on the encapsulation efficiency and the effect of the molar ratio of PEG2000-COOH to MPEG2000 on the Tf connection rate, respectively.

As shown in Figure 5A, the encapsulation efficiency (EE) reached its peak when the molar ratio of total MPEG to phospholipids was set at 5%. As observed in Figure 5B, the connection rate of the Tf peaked when the molar ratio of DSPE-PEG2000-COOH to DSPE-MPEG2000 reached 10% and subsequently declined with further increases in the ratio.

### 2.5. Characterization of the Liposomes

**Figure 6:**
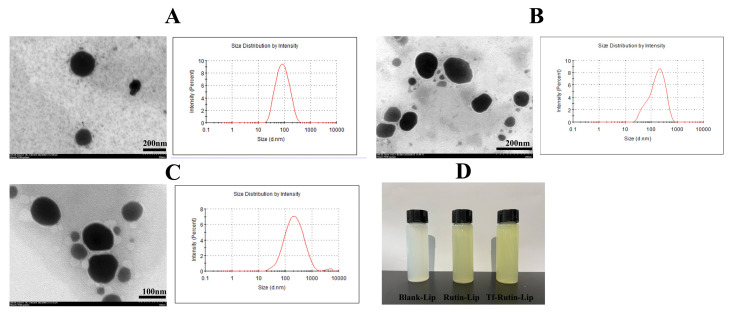
Characterization of three nanoparticles. (**A**) Transmission electron micrographs of blank liposomes and the corresponding particle size distribution. (**B**) Transmission electron micrographs of Rutin-Lip and the corresponding particle size distribution. (**C**) Transmission electron micrographs of Tf-Rutin-Lip and the corresponding particle size distribution. (**D**) Appearance of nanoliposome solutions.

The TEM images (Figure 6) revealed that the liposomes were generally spherical and of regular size. The appearance of Rutin-Lip and Tf-Rutin-Lip was homogeneous, transparent, and pale yellow. Physical properties, including the particle size, polydispersity index (PDI), zeta potential, encapsulation efficiency, drug loading capacity, and Tf grafting rate, are exhibited in Table 3. The EE% of Rutin-Lip and Tf-Rutin-Lip were 70.77 ± 0.645% and 72.68 ± 0.035%, respectively. The mean particle sizes of blank liposomes, Rutin-Lip, and Tf-Rutin-Lip were 88.8 ± 1.15, 112.4 ± 0.30, and 132.8 ± 1.563, respectively. The polydispersity indices, which represent the distribution of the particle sizes, were 0.29 ± 0.044, 0.371 ± 0.009, and 0.398 ± 0.021, respectively, with potentials of 0.578 ± 0.234, 0.694 ± 0.649, and 0.787 ± 0.407, and the grafting rate of the Tf in Tf-Rutin-Lip was 54.32 ± 0.067%.

### 2.6. In Vitro Release Study

**Figure 7:**
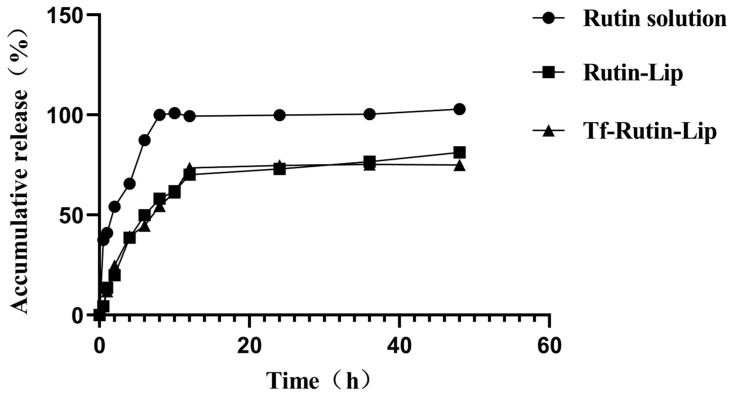
Cumulative release percentage of each sample in vitro (Mean ± SD, *n* = 3).

The in vitro release profiles of Rutin, Rutin-Lip, and Tf-Rutin-Lip are illustrated in Figure 7. The results indicate that the Rutin solution group exhibited a rapid release, with a cumulative release percentage of 100% within eight hours. However, the liposomes displayed a slower release rate compared to the Rutin solution group. Specifically, approximately 58.29% and 54.59% of Rutin was released from the Rutin-Lip and Tf-Rutin-Lip groups within 8 h. By 48 h, the cumulative release of the Rutin-Lip and Tf-Rutin-Lip groups reached 81.32% and 75.12%, respectively. 

### 2.7. Preliminary Evaluation of the Two Nanoformulations Passing Through Blood–Brain Barrier Model

**Figure 8:**
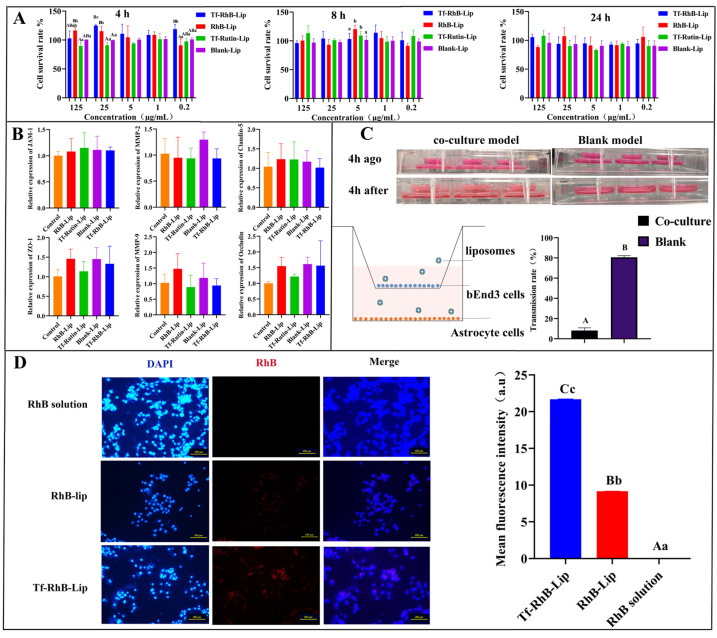
Evaluation of different liposomal formulations for brain targeting. (**A**) The effect of different concentrations (125, 25, 5, 1, and 0.2 μg/mL) of liposomes on the viability of bEnd.3 cells. (**B**) The effect of liposomes on the integrity of the blood–brain barrier. Real-time quantitative polymerase chain reaction analysis was used to detect the mRNA relative expression of *JAM*-1, *ZO*-1, *MMP*-2, *MMP*-9, *Claudin*-5, and *Occludin* of bEnd.3 cells. (**C**) The pattern mapping and results of functional test of in vitro BBB model. An in vitro BBB model was established using bEnd.3 cells and C8-D1A astrocytes. A four-hour leakage assay and a fluorescein sodium permeability assay were used to verify the BBB model. The BBB model was considered successful when the results of the 4 h leak test showed no change in altitude difference, and there was no fluorescent signal from the lower chamber. (**D**) Analysis of fluorescence intensity of in vitro BBB model incubated with the varying liposomal formulations. An amount of 5 µg/mL free RhB solution, RhB-Lip, and Tf-RhB-Lip was added to the upper chamber of BBB model and cultured for 4 h at 37 °C (*n* = 3), the fluorescence intensity of RhB and DAPI in the lower chamber was observed using fluorescence microscopy. Scale bar = 100 μm. Mean fluorescence intensity was analyzed with ImageJ. Between groups, different uppercase letters indicate extremely significant differences (*p* < 0.01), different lowercase letters indicate significant differences (*p* < 0.05), and the same letter markings indicate no significant differences (*p* > 0.05).

The result of different concentrations of liposomal formulations (125, 25, 5, 1, and 0.5 μg/mL) on the viability of bEnd.3 cells is shown in Figure 8A. After 4 h of incubation, the cell viability exceeded 90% for all formulations except for Tf-Rutin-Lip at a concentration of 125 μg/mL, which exhibited a cell viability below 90%. At the 8 h incubation period, cell viability remained greater than 90% for all experimental preparations. After 24 h, RhB-Lip at a concentration of 125 μg/mL and Tf-Rutin-Lip at a concentration of 5 μg/mL demonstrated cell viability below 90%, whereas the remaining formulations maintained cell viability above 90% across all tested concentrations. In summary, the evaluated drugs exhibited minimal impact on cell viability within the concentration range of 125 μg/mL to 0.2 μg/mL, suggesting a favorable safety profile at these concentrations. Upon conducting real-time quantitative polymerase chain reaction (qPCR) analysis, we observed a notable upregulation of *JAM-1*, *Claudin-5*, *ZO-1*, and *Occludin* expression levels in RhB-Lip, Tf-Rutin-Lip, Blank-Lip, and Tf-RhB-Lip; in contrast, the expression of *MMP-2* and *MMP-9* was markedly downregulated in these same groups (Figure 8B). The results showed that the four agents did not disrupt the tight junction of the blood–brain barrier and demonstrated the feasibility of establishing the blood–brain barrier.

The in vitro blood–brain barrier (BBB) model was evaluated using a four-hour leakage test and a fluorescein sodium permeability test. As demonstrated by the four-hour leakage test, the BBB model maintained its liquid level difference after four hours, indicating that the requirements for a functional BBB model were met in vitro (Figure 8C). The results of the sodium fluorescein permeability test showed lower permeability in the in vitro BBB model (Figure 8C). Fluorescence microscopy was used to observe the fluorescence intensity of cells in the underlying pores of the in vitro BBB model, where RhB dye was substituted for Rutin (Figure 8D). After adding the RhB solution to the upper chamber and incubating for two hours, no red fluorescence was observed in the lower chamber cells, indicating that RhB solution alone could not traverse the BBB model in vitro. This suggests that essential medications alone have difficulty penetrating the BBB and reaching the brain. However, under identical conditions, very faint red fluorescence was observed in the RhB-Lip group, indicating that the liposomal phospholipid bilayer structure played a significant role. This structure closely resembles the plasma membrane of human cells, thus exhibiting good biocompatibility. The fluorescence intensity was most pronounced in the Tf-RhB-Lip group, with stronger fluorescence observed in the lower chamber cells, significantly surpassing the other two groups. Due to the presence of TfR on brain endothelial cells, when liposomes are decorated with Tf on their surface, they gain the ability to traverse the blood–brain barrier (BBB) more efficiently and enhance accumulation in the underlying cells.

**Figure 9:**
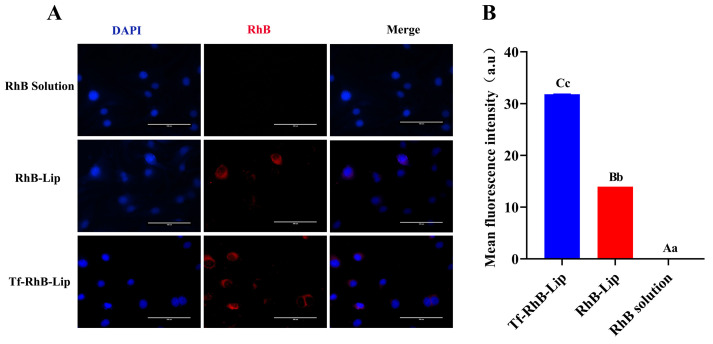
Cellular uptake and fluorescence intensity analysis after incubation with different formulations. (**A**) Analysis of fluorescence intensity of bEnd.3 cells incubated with the varying liposomal formulations using fluorescence microscopy; scale bar = 100 μm. (**B**) Mean fluorescence intensity of bEnd.3 cells incubated with the varying liposomal formulations. Between groups, different uppercase letters indicate extremely significant differences (*p* < 0.01), different lowercase letters indicate significant differences (*p* < 0.05), and the same letter markings indicate no significant differences (*p* > 0.05).

In accordance with existing studies on its endocytic mechanism, Rutin was replaced by RhB dye to determine the intracellular uptake and distribution of the liposome carriers. Red fluorescence was clearly observed in Tf-RhB-Lip, and was much stronger than that observed in RhB-Lip. The fluorescence intensity analysis showed that the fluorescence intensity of Tf-RhB-Lip was significantly higher than that of RhB-Lip, suggesting that Tf-RhB-Lip exhibited better penetration in bEnd.3 cells (Figure 9).

**Figure 10:**
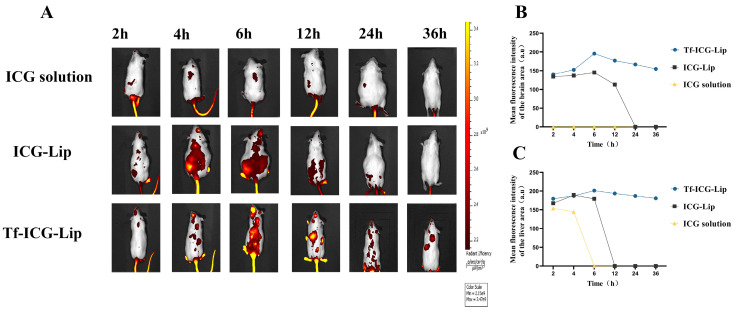
(**A**) Real-time imaging observation of Kunming mice after intravenous injection of different liposome formulations. (**B**) Analysis of fluorescence intensity in the brain of Kunming mice after intravenous injection of different liposome formulations. (**C**) Analysis of fluorescence intensity in the liver of Kunming mice after intravenous injection of different liposome formulations.

ICG dye was used as a substitute for Rutin to observe the brain-targeting capability of the liposomes. The three different formulations, ICG solution, ICG-Lip, and Tf-ICG-Lip, exhibited distinct effects on brain targeting capacity (Figure 10A). All groups showed varying fluorescence intensities from 2 h to 6 h, with the ICG solution group displaying very low fluorescence intensity and no fluorescence in the brain compared to the ICG-Lip and Tf-ICG-Lip groups, despite peaking at 6 h. This observation further confirms that conventional drugs cannot penetrate the BBB in vivo. In comparison to the ICG solution group, the ICG-Lip group exhibited a more intense fluorescence intensity. It displayed some fluorescence in the brain, indicating that the liposome structure plays a role in penetrating the BBB (Figure 10A).

In contrast to the other two groups, the Tf-ICG-Lip group exhibited the most robust fluorescence intensity in the brain and sustained the signal for up to 36 h (Figure 10B). This clearly demonstrates that Tf-modified liposomes possess significantly enhanced brain-targeting ability. The prolonged in vivo metabolism of Tf-ICG-Lip may be attributed to the use of long-circulating materials, which extend its circulation time within the bloodstream. Nonetheless, the comparison between groups underscores the remarkable enhancement in the fluorescence signal of brain tissue following Tf modification, thus partially explaining its brain-targeting capability.

### 2.8. Specificity, Linearity, LOD, and LOQ

**Figure 11:**
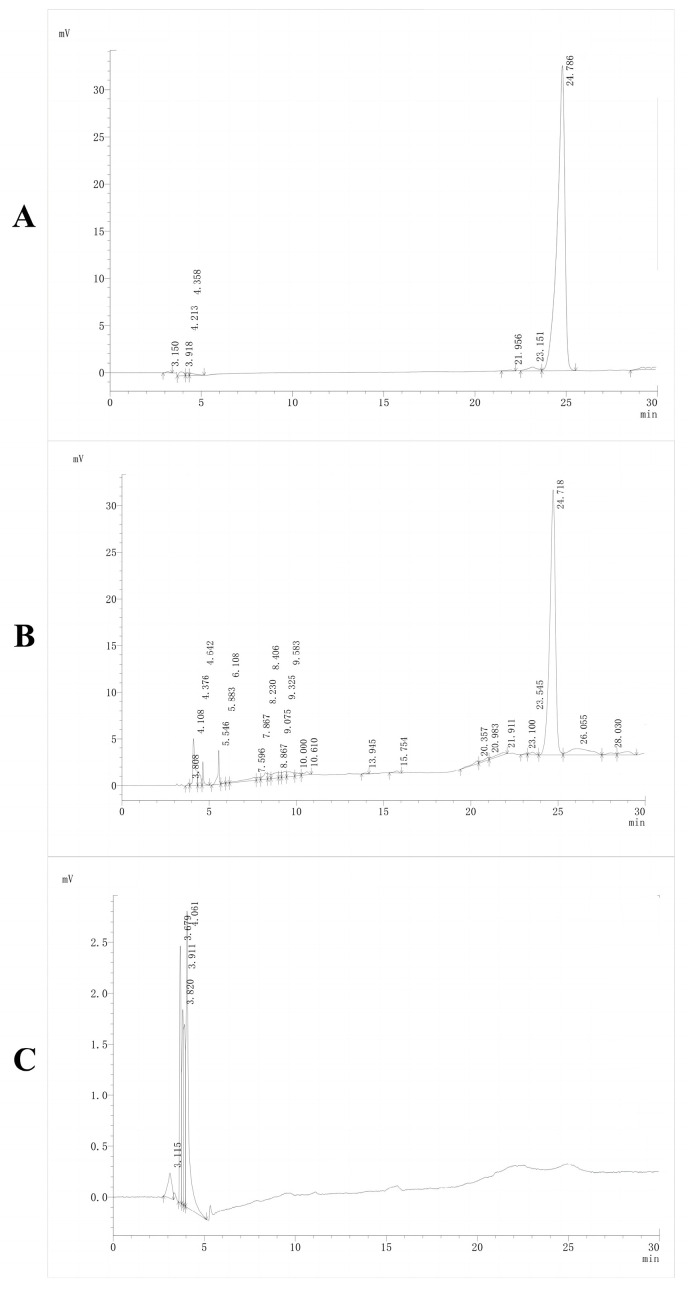
HPLC chromatogram of rat plasma samples. (**A**) Rutin standard (10 µg/mL) chromatograms. (**B**) Chromatograms of blank plasma samples spiked with Rutin. (**C**) Chromatograms of blank plasma. Chromatographic separation was carried out on an Intertsil ODS-3 C18 column (250 mm × 4.6 mm, 4 µm) using a Shimadzu 2050A liquid chromatograph. The analysis was performed with ultraviolet (UV) detection using a diode array detector (DAD) set at 358 nm for Rutin. The mobile phase consisted of methanol and 0.5% H_3_PO_4_ (50:50 *v*/*v*). The flow rate was set at 0.8 mL/min, the injection volume was set at 20 μL, and the column temperature was set at 30 °C. The Rutin retention time was found to be 24.78 min.

The peaks of the Rutin target chromatograms were symmetrical and without trailing and separated well from endogenous substances in plasma (Figure 11). The linearity of the Rutin was assessed for various plasma standard solutions over the range of 0.01–5 μg/mL. The weighted least-squares linear regression was fitted over the 0.01–5 μg/mL range for Rutin. Rutin’s calibration curve exhibited excellent linearity and a high correlation coefficient. The linear regression equation was y = 73,576x − 1828.5 (where y is peak area, x is Rutin concentration, and wi = 1/x) with a correlation coefficient of 0.9998. The LOD and LOQ values were 0.01 and 0.05 ng/mL, respectively.

### 2.9. Recovery Rate Results

The accuracy and precision were expressed as recovery and coefficient of variation, respectively. Rutin standard solutions at three different concentrations were added to plasma samples containing known quantities of Rutin. Then, the Rutin concentration of the plasma samples was determined, and the recovery and coefficient of variation of the added Rutin were calculated. The recovery and coefficient of variation of Rutin in plasma samples at three different spiked concentrations of 0.1, 1, and 5 µg/mL are shown in Table 4. The average recovery of Rutin in plasma ranged from 91.82% to 101.18%, with an inter-batch coefficient of variation within 6.60% and an intra-batch coefficient of variation of less than 8.48%.

### 2.10. Stability Results

To evaluate the stability of Rutin in rat plasma, the QC samples in three complete freeze–thaw cycles from −80 °C to room temperature and long-term storage at −80 °C for 30 days were assessed. Table 5 indicates that after the freeze–thaw cycles, the mean concentrations of the samples at concentrations of 0.1, 1, and 5 μg/mL were 0.0967, 1.0042, and 4.2922 μg/mL, respectively, with accuracies of 96.76%, 100.42%, and 85.85%. Following long-term freezing, the average concentrations for the same samples were 0.0966, 0.9555, and 4.4929 μg/mL, respectively, exhibiting accuracies of 96.67%, 95.56%, and 89.86%. No significant changes were observed under these conditions, suggesting good stability.

### 2.11. Pharmacokinetic Parameters

**Figure 12:**
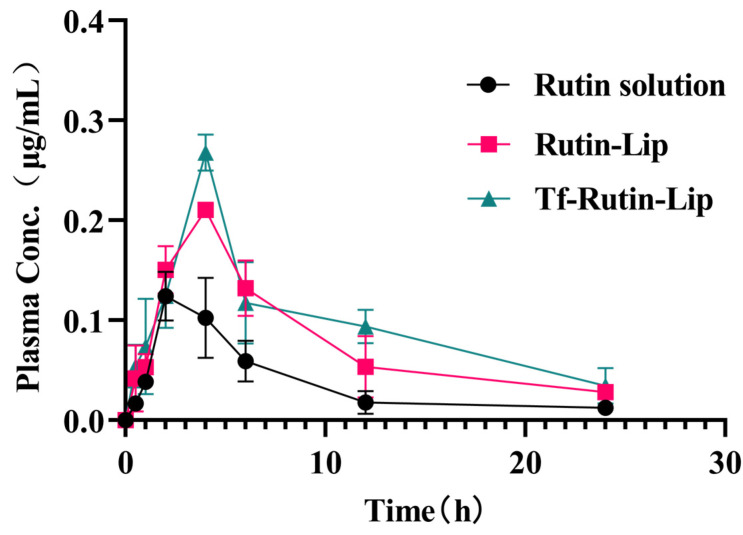
Plasma concentration time curves of Rutin in SD rats at different time points.

To evaluate the pharmacokinetic properties, the pharmacokinetics of free Rutin solution, Rutin-Lip, and Tf-Rutin-Lip were evaluated after oral gavage of an equivalent dose of 100 mg/kg Rutin. The plasma concentration time profiles are presented in Figure 12. The pharmacokinetic parameters were measured using WinNonlin software (version 8.1.0.3530) and are shown in Table 6. The results demonstrated that Tf-Rutin-Lip prominently improved drug exposure, with the area under the curve (AUC_0-t_) increasing from 0.901 ± 0.187 µg·h/mL to 2.324 ± 0.212 µg·h/mL. Moreover, compared with the free Rutin solution, the AUC _0-t_ of Rutin-Lip and Tf-Rutin-Lip increased 2.09-fold and 2.58-fold, respectively. Likewise, AUC_0-∞_and C_max_ increased 2.77-fold and 1.30-fold (*p* < 0.01) for Tf-Rutin-Lip compared to free Rutin solution and Rutin-Lip, respectively. Furthermore, compared with the free Rutin group, the plasma clearance (CL) markedly decreased (*p* < 0.01), and the mean residential time (MRT_0-∞_) of Tf-Rutin-Lip significantly increased (*p* < 0.01). The plasma CL of free Rutin solution in rats was significantly higher than that of Rutin-Lip and Tf-Rutin-Lip, revealing the rapid removal of Uro-A from the body and reducing the remaining quantity of residual drugs.

It showed that liposomes could increase the MRT and biological half-life of Rutin in plasma and its bioavailability. In addition, Tf-Rutin-Lip showed prolonged half-life (T_1/2_), decreased clearance (CL), and increased area under the curve (AUC_0-t_), and these results suggest that liposome surfaces modified with Tf and polyethylene glycol may prolong their mean residence time to achieve a delayed-release effect (Figure 12).

## 3. Discussion

In the presence of the blood–brain barrier (BBB), over 98% of drugs face challenges in penetrating the brain, significantly reducing their therapeutic efficacy against various brain diseases. To circumvent this obstacle, innovative nanotargeting technologies have emerged as a promising approach to enhance drug concentration and therapeutic action at the target site. These nanosystems effectively facilitate the transport of drug-active ingredients across the BBB through encapsulation or by targeting and modifying BBB-specific ligands on their surfaces [33]. In our study, we successfully developed a transferrin (Tf)-modified liposome that demonstrates the ability to traverse the BBB. This nanosystem encapsulates Rutin, a flavonoid with numerous biological activities, including anti-inflammatory, antioxidant, neuroprotective, nephroprotective, and hepatoprotective effects. Moreover, Rutin has garnered significant attention for its potential therapeutic applications in treating several brain disorders. Specifically, it has been investigated as a potential treatment for Alzheimer’s disease [34], exhibits neuroprotective properties by mitigating oxidative and pro-inflammatory brain damage [35], possesses anti-glioblastoma activity [36,37], ameliorates cerebral ischemia–reperfusion injuries, and reduces brain damage through its antioxidant and anti-inflammatory mechanisms [38]. The Tf modification on our liposome is particularly significant given the high expression of TfR in cerebrovascular epithelial cells. This modification enables the liposome to specifically target the brain and enhance its capacity to traverse the BBB, ultimately leading to improved therapeutic outcomes in the treatment of brain diseases.

In this study, we preferred the classic preparation method of liposomes, the thin-film dispersion method, to prepare brain-targeting liposomes [26]. First, we prepared Rutin liposomes, and the optimal preparation conditions were obtained by response surface testing. Then, we modified the surface of the Rutin liposomes with Tf using covalent bonding, thus obtaining the Tf-modified Rutin liposomes. The choice of preparation method is particularly important for the preparation of liposomes, and different methods will have different effects on the particle size and EE% of the liposomes. Various methods have been developed to prepare nanoliposomes, which can be selected according to the nature of different drugs. The reverse evaporation method is suitable for hydrophilic drugs, whereas the film dispersion method is typically chosen for lipophilic drugs [39]. In the preliminary tests, we screened the various liposome preparation methods to determine the most suitable approach. The ethanol injection method involves settling during the volatilization of ethanol and cannot form liposomes. Moreover, the reverse evaporation method is suitable for hydrophilic drugs, but organic solvents cannot evaporate cleanly during the preparation process, leading to a decrease in the liposome EE%. Therefore, we chose the thin-film dispersion method for the preparation of our liposomes.

In a single-factor test, the EE decreased when the lipid-to-cholesterol ratio exceeded 4:1. This decrease in EE may be due to the imbalance between the cholesterol and soy lecithin content. In addition, the drug-to-lipid ratio was minimized, with the EE% at 1:60. This decline could be attributed to the increased mobility and reduced stability of the liposomes, likely due to the higher soy lecithin content, resulting in depolymerization and drug leakage. The highest EE% was achieved when the preparation temperature reached 45 °C. This optimal temperature may be conducive to the arrangement of the phospholipid bilayers and the interactions between Rutin and the phospholipids. However, beyond 45 °C, the EE% decreased, likely due to the disruption of the membrane structure from the high temperatures, resulting in drug leakage from the liposomes. When the surfactant content was increased from 80 mg/mL to 100 mg/mL, the EE% decreased significantly. This was likely due to Tween 80, a nonionic hydrophilic surfactant with long hydrophilic and hydrophobic chains, which may have interfered with the formation and stability of the liposomes, resulting in a decline in EE. Excessive sonication may cause the rupturing of the lipid film, leading to the leakage of Rutin and a corresponding decrease in EE. The volume of the aqueous phase increases from within a certain range, and the mobility and range of Rutin and phospholipids are improved, increasing the capture rate of phospholipids on the core material and, thus, increasing the EE.

The PEGylation methods for liposomes are classified into two types: the one-step method and the post-insertion method [28]. The one-step method involves forming membranes of long-circulating materials with phospholipids, cholesterol, and other membrane components to produce long-circulating liposomes. Alternatively, the post-insertion method first prepares conventional liposomes and then introduces DSPE-PEG2000 into these liposomes for incubation. This process utilizes the interaction between the hydrophobic lipid moiety of DSPE-PEG2000 and the phospholipids on the liposome membrane surface, allowing the self-assembly of DSPE-PEG2000 to partially insert into the phospholipid bilayer of the liposome, thus forming a long-circulating liposome. With the post-insertion method, the active groups on the DSPE-PEG2000 are fully exposed on the surface of the liposomes, effectively enhancing the coupling efficiency with targeted ligands. Therefore, the post-insertion method was chosen for the preparation of brain-targeted liposomes, which not only extends the in vivo circulation time of the liposomes, but also avoids phagocytosis by liver cells. Surface modification with transferrin (Tf) was then performed by attaching the amino group of Tf to the liposome surface using EDC and NHS as coupling agents. The Tf modification was subsequently identified and confirmed using a BCA kit. Studies have also demonstrated the advantages of Tf-modified liposomes [40,41]. For instance, Sonali et al. [40] prepared Tf-modified d-alpha-tocopherol polyethylene glycol 1000 succinate mono-ester (TPGS) liposomes and found that the in vitro release was sustained for over 72 h, achieving a release rate of 71%. Similarly, Wang et al. [41] developed transferrin-modified dioscin-loaded PEGylated liposomes, which exhibited approximately 30% sustained release over 72 h at 37 °C. In the current study, Tf-Rutin-Lip exhibited a release rate of 54.59% for Rutin within eight hours and achieved 75.12% release by 48 h. Additionally, it displayed a slow-release profile in pharmacokinetic assessments.

After successfully synthesizing brain-targeted liposomes, we evaluated their brain targeting efficiency through in vivo and ex vivo experiments. To validate the in vitro blood–brain barrier (BBB) model, we substituted RhB for Rutin in our assays. Rhodamine B is a significant fluorescent dye that serves as a cellular fluorescent stain in biological and medical research, commonly used for cell staining and labeling. Its superior spectral characteristics, good biocompatibility, moderate molecular weight, ease of manipulation, and wide range of applications make RhB one of the essential tools for studying the permeability of the blood–brain barrier (BBB) and drug delivery mechanisms [42]. Regarding cell selection, the co-culture of endothelial cells and glial cells is a common approach to constructing a BBB model. Consequently, we employed bEnd.3 cells and astrocytes to mimic the BBB. It has been previously demonstrated that the co-culture of vascular endothelial cells and astrocytes mitigates the “phenotypic drift” observed in monolayer cell models, thereby preserving the integrity of the BBB. *JAM-1*, *Claudin-5*, and *ZO-1*, as crucial proteins of tight junctions, are vital for maintaining the integrity and permeability of the BBB. Meanwhile, the matrix metalloproteinases *MMP-2* and *MMP-9* regulate BBB permeability under pathological conditions, whereas Occludin further controls the intercellular permeability of the BBB through homophilic interactions. Together, these molecules ensure that the BBB effectively protects the CNS [43].

Chen et al. successfully established an in vitro BBB model using bEnd.3 cells and astrocytes, which can effectively demonstrate the permeability of drugs. Furthermore, RhB was used as a substitute for α-mangostin to determine the intracellular distribution of liposome carriers at different time points. The results showed that the transferrin-conjugated liposomes exhibited intense and clear fluorescence [44]. Antonio Lopalco et al. [45] found that Tf-modified liposomes traverse the in vitro BBB more efficiently compared to conventional liposomes. In our study, the integrity of the BBB model was assessed through a 4 h leakage test and a fluorescein sodium permeability test, confirming that our model met the fundamental permeability criteria. In the in vitro experiment on BBB penetration, we followed the method described by Chen but substituted RhB for rutin. Our results indicated that the Tf-RhB-Lip group displayed greater fluorescence accumulation in the lower cell layer compared to the RhB solution group and the RhB-Lip group, suggesting an enhanced ability of Tf-modified liposomes to traverse the BBB. Among them, the RhB-Lip group showed slight fluorescence, which may be attributed to the enhanced interaction with the cell membrane by RhB-Lip attaching to the cell surface through physical adsorption and lipid exchange. Subsequently, the cell may phagocytose the liposomes as foreign objects through endocytosis, encapsulate them within vesicles, and then transport them to lysosomes for digestion. Within the lysosome, the liposome membrane structure is degraded, thereby releasing the encapsulated drug or other active substances. Additionally, liposomes have the potential to fuse directly with the cell membrane, releasing their contents directly into the cell interior [46].

In our in vivo study, we utilized ICG as a surrogate for Rutin, as ICG is currently the only clinically approved fluorescent probe. It emits fluorescence at a specific wavelength when excited by a corresponding light source, enabling fluorescence imaging in vivo and ex vivo through optical imaging devices. This allows for the differentiation of labeled substances or tissues from their surroundings [47]. Portnoy’s research has unveiled that encapsulating ICG within liposomes significantly enhances its emission intensity, particularly manifesting in a more pronounced signal emission within the brains of infected mice [48]. This discovery underscores the pivotal potential of ICG-loaded liposomes in the diagnosis and therapeutic monitoring of cerebral malaria, thereby validating the highly feasible and valuable selection of ICG as an effective biomarker. This study observed the accumulation of ICG in all three groups: ICG solution, ICG-Lip, and Tf-ICG-Lip. Notably, a significant enhancement in ICG accumulation was observed in the brain with the application of Tf modification. Similarly, in a study conducted by Liang Kong, Osthole (Ost) was substituted with DiR, and various preparations were examined, including Ost solution, Ost-Lip, and Tf-Ost-Lip. Among these, Tf-modified liposomes exhibited robust fluorescence in the brain, with significantly higher accumulation compared to the other two groups [49]. Further, a study by Bruna dos Santos Rodrigues found that Tf-modified liposomes possess superior fluorescence intensity in the brain [50]. Xiao-Li Song et al. [51] also corroborated this finding, demonstrating that Tf-modified polyethylene glycol liposomes enhanced trans-BBB transport. In vivo imaging results revealed that the fluorescent signal was sustained in the brain for up to 48 h. Conversely, upon administration of free DiR fluorescent dye, the fluorescent signal was rapidly distributed to the liver and gradually diminished or disappeared after 24 h. These results align with our findings, indicating that Tf modification facilitates liposome accumulation in the brain and underscores the targeting ability of Tf towards the brain.

We conducted pharmacokinetic testing in plasma to determine the Rutin content. High-performance liquid chromatography (HPLC) was selected for this purpose, as it is a rapid separation and analytical technique characterized by high separation efficiency, low detection limits, automated operation, and broad applicability. An Intertsil ODS-3 column (250 mm × 4.6 mm, 5 μm) was employed. Initially, an isocratic elution method using a mixture of 50% methanol and 50% 0.5% aqueous phosphoric acid was attempted, but this resulted in poor separation and peak shapes. Subsequently, a gradient elution method was implemented, which significantly improved the separation ability and peak shapes, with minimal trailing. Additionally, gradient elution shortened the analysis cycles and increased sensitivity, albeit with the occasional occurrence of baseline drift. In this study, the drug was administered through oral gavage, which offers the advantages of precise drug delivery and dosage control. After oral administration and entry into the small intestine, the liposome is dispersed into tiny oil droplets by bile, which are further broken down into small molecular lipids by pancreatic enzymes. Subsequently, bile salts bind with lipid molecules to form complexes, facilitating their absorption on the microvilli of the small intestine. Inside the microvillus cells, the lipids are reassembled into chylomicrons. These chylomicrons are processed by cellular organelles and released into the intestinal lumen, ultimately entering the bloodstream via the lymphatic system [52,53]. In selecting the oral gavage dose, we tested various doses (such as 50 mg/kg, 100 mg/kg, and 200 mg/kg) of the drug in rats to observe their physiological responses and pharmacokinetic parameters. The results indicated that the 100 mg/kg dose provided sufficient drug exposure, with no apparent toxicity being observed; therefore, it was chosen as the dose for further investigation. Additionally, considering the solubility, stability, absorption rate, and anticipated pharmacodynamic potency of the drug, the 100 mg/kg dose was deemed to offer an appropriate concentration range, enabling the accurate assessment of its pharmacokinetic behavior.

The pharmacokinetic parameters in the rat plasma revealed that the Rutin-Lip and Tf-Rutin-Lip groups enhanced the bioavailability of Rutin compared to the Rutin solution group. Notably, the Tf-Rutin-Lip group exhibited a more pronounced increase in the elimination half-life (T_1/2_), a decrease in clearance (CL), and an increase in the area under the curve (AUC_0-t_). This enhancement may be attributed to the long-circulating nature of Tf-Rutin-Lip, modified with long-circulating substances, which may reduce phagocytosis and absorption by the reticuloendothelial system (RES), ultimately prolonging drug circulation time and improving bioavailability. Particle size is a crucial factor influencing the passage of liposomes through the intestinal mucus layer and across intestinal cells [54]. Immunoliposomes, specifically, typically have a particle size below 200 nm. In this study, we prepared Rutin-Lip and Tf-Rutin-Lip with particle sizes below 140 nm, favoring the intracellular transport of Rutin.

## 4. Materials and Methods

### 4.1. Materials

Rutin (purity ≥ 98%), phosphate-buffered saline (PBS, pH 7.4), N-hydroxybutanediimide (NHS, purity > 99%), gelatin aqueous solution (1%), and dialysis cassettes with an 8 kDa to 14 kDa molecular weight cutoff (MWCO) were provided by Beijing Solarbio Science & Technology Co., Ltd. (Beijing, China). Soy lecithin (SPC, purity ≥ 98%), cholesterol (CH, purity ≥ 99%), DSPE-MPEG2000 (purity ≥ 99%), and DSPE-PEG2000-COOH (purity ≥ 95%) were supplied by AVT (Shanghai) Pharmaceutical Tech Co., Ltd. (Shanghai, China). Transferrin (Tf, purity > 98%) was purchased from Shanghai Yuanye Bio-Technology Co., Ltd. (Shanghai, China). Millipore ultrafiltration centrifuge tubes were purchased from Merck (MWCO: 3 kDa; Shanghai, China). 1-ethyl-3-(3-dimethylamino propyl) carbodiimide hydrochloride (EDC, purity > 98%), sodium dodecyl sulfate (SDS), sodium fluorescein, and high-performance liquid chromatography (HPLC)-grade methanol (purity ≥ 99.9%) were obtained from Shanghai Aladdin Biochemical Technology Co., Ltd. (Shanghai, China). The HPLC chromatographic Inertsil ODS-3 column (4.6 × 250 mm, 5 μm) was purchased from GL Science (Tokyo, Japan). Fetal bovine serum (FBS), Dulbecco’s modified eagle medium (DMEM), penicillin, streptomycin, trypsin-EDTA, and dimethyl sulfoxide (DMSO) were purchased from Gibco (Gibco, Shanghai, China). 4′,6-Diamidine-2′-phenylindole dihydro-chloride (DAPI), rhodamine B isothiocyanate (RhB), and Indocyanine Green (ICG) were obtained from Shanghai Macklin Biochemical Technology Co., Ltd. (Shanghai, China). Other chemicals used were of analytical grade.

### 4.2. Animals

Female Sprague Dawley (SD) rats (200 ± 20 g) and Kunming mice (20 ± 2 g) were supplied by Tianqin Biotechnology Co., Ltd. (Changsha, China). The animals were maintained at a temperature of 20 °C ± 2 °C, on a 12 h light/12 h dark cycle, and relative humidity of 50–60%, with free access to food and water. The rats were fasted for 12 h before oral administration. All procedures involving animals were performed by Institutional Animal Care guidelines and approved by the Animal Ethics Committee of Guangxi University (Nanning, China; certification number: GXU-2022-210).

### 4.3. Cell Culture

Mouse brain microvascular endothelial cells (bEnd.3) (Procell, Wuhan, China) and the mouse C8-D1A astrocyte cell line, a kind gift from Professor Xiao-Ning Li, were all cultured in high glucose (4.5 g/L)-containing DMEM with 10% FBS, 100 U/mL penicillin, and 100 μg/mL streptomycin. The cells were incubated at 37 °C in a 5% CO_2_-95% air humidified incubator. All experiments were performed in the logarithmic phase of cell growth.

### 4.4. Single-Factor Design

Six major single factors that affect Rutin encapsulation efficiency were selected for further experiments via a single-factor design. The experimental conditions were set as follows: weight ratios of phospholipid to cholesterol (X1) were designed from 4:1 to 12:1, weight ratios of drug to phospholipid (X2) were designed from 1:3 to 1:15, the preparation temperatures (X3) were designed from 35 °C to 65 °C, the volume of PBS (X4) was prescribed from 5 mL to 25 mL, sonication times (X5) from 5 to 25 min were used, and Tween 80 concentrations (X6) were set from 2 to 10 mg/mL. In each experiment, only one factor was changed at a time while the others were kept constant.

### 4.5. Design of Experiments (DOE)

A three-level, three-factor Box–Behnken design (BBD) was employed to evaluate the individual and combined effects of variables on liposome encapsulation efficiency. The dependent variable was the encapsulation efficiency percentage (EE%) (Y). The phospholipid-to-cholesterol ratio (%, *w*/*w*), drug-to-lipid ratio (%, *w*/*w*), and preparation temperature (°C) were selected as three independent variables based on the results from the single-factor experiments (Table 7). A total of 17 combinations were designed and analyzed using Design Expert^®^ (version 12.0, Stat-Ease Inc., Minneapolis, MN, USA). Details of the experimental design for these formulations are indexed in Table 1. A quadratic model equation was generated between the factors and responses, and is described as follows:

Y = b0 + b1A + b2B + b3C + b12AB + b11A2 + b22B2 + b33C2

where Y represents the dependent variable; b0 is the model constant; b1, b2, and b3 are linear coefficients; b12, b13, and b23 are interaction coefficients; b11, b22, and b33 are quadratic coefficients; and A, B, and C are the coded levels of independent variables. The terms AB, AC, and BC and A2, B2, and C2 represent the interaction and quadratic terms, respectively. 

The optimization of Rutin-Lip was performed using an analysis of variance (ANOVA) in Design Expert^®^ 12.0 through the simultaneous fitting of the observed responses to linear, 2FI, quadratic, and cubic models, based on the statistical significance of the coefficients and R^2^ values. The optimum values of the independent factors were selected based on the desirability criteria of maximum encapsulation efficiency.

### 4.6. Preparation of Rutin-Lip and Tf-Rutin-Lip

Rutin-Lip was prepared using the thin-film hydration method with modifications [8]. In brief, specific amounts of Rutin (3 mg), SPC (150 mg), CH (37.5 mg), and Tween 80 (80 mg/mL) were dissolved in 20 mL of methanol. Then, the mixtures were evaporated at 45 °C in a rotary evaporator to remove the traces of solvent until film formation was observed. The film was hydrated with 0.01 mol/L phosphate-buffered solution (PBS, pH 7.4, 20 mL). The vesicle suspension was then crushed for 10 min using a probe sonicator and filtered through a 0.45 µm syringe filter (Millipore 33 mm). The Rutin-Lip suspensions were stored at 4 °C until use.

**Figure 13:**
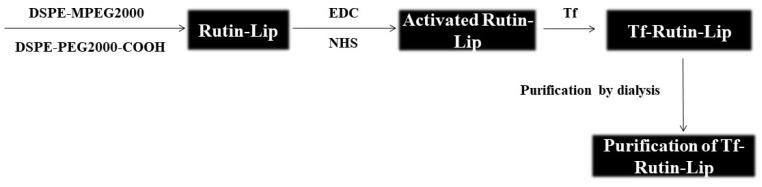
Schematic representation of the preparation of Tf-Rutin-lip.

Tf-Rutin-Lip was prepared by attaching Tf to the surfaces of Rutin-Lip through a two-step EDC–NHS coupling method. The schematic representation of the preparation of Tf-Rutin-lip is shown in Figure 13. Briefly, liposomes were incubated with DSPE-MPEG2000 (the molar ratios of DSPE-MPEG2000 to soy lecithin were 4%, 5%, 6%, 7%, and 8%) and DSPE-PEG2000-COOH (the DSPE-PEG2000-COOH to DSPE-MPEG2000 molar ratios were 3%, 5%, 7%, and 9%) at 60 °C for one hour. An EDC–NHS catalytic solution (10:10:1 molar ratio of EDC, NHS, and DSPE-PEG2000-COOH) was added to the Rutin-Lip suspension, and the mixture was stirred at 100 rpm for 15 min at room temperature. The Tf aqueous solution was added, and the molar ratio between DSPE-PEG2000-COOH and Tf was 40:1. The Tf-Rutin-Lip suspension was obtained after 3 h of stirring. The liposome suspension was subsequently dialyzed against PBS (pH 7.4) under gentle stirring (250 rpm) for the next 6 h. The dialysis medium was changed once every three hours. The dialyzed liposome suspension was collected and stored at 4 °C until use. The RhB-loaded liposomes (Tf-RhB-Lip, RhB-Lip), ICG-loaded liposomes (Tf-ICG-Lip, ICG-Lip), or blank liposomes were similarly prepared according to the above methods, with Rutin replaced by RhB/ICG or removed directly.

### 4.7. Characterization of Liposomes

#### 4.7.1. Morphology

The morphology of Rutin-Lip and Tf-Rutin-Lip was observed using transmission electron microscopy (TEM, HITACHI HT7700, Tokyo, Japan). The liposome suspensions were diluted with deionized water and dropped onto a carbon-coated copper grid (200-mesh), and, after staining with a phosphotungstic acid solution (2%, *w*/*v*, pH 7.0), the excessive liquid was removed with filter paper. The air-dried samples were then observed under an 80 kV accelerating potential electron beam via TEM.

#### 4.7.2. Particle Size, PDI, Zeta Potential and Tf Grafting Rate

The particle size, polydispersity index (PDI), and zeta potential of the Rutin-Lip and Tf-Rutin-Lip suspensions were measured using a Zetasizer-Nano-ZS90 (Malvern instruments, Malvern, UK). The analyses (*n* = 3) were carried out for 100 s at 25 °C.

The Tf grafting rate on the surface of the liposomes was indirectly determined using a bicinchoninic acid (BCA) protein quantification kit [55]. The Tf-Rutin-Lip suspension was centrifugated at 10,000 rpm for 30 min to remove free Tf. Thereafter, methanol and chloroform were added to the Tf-Rutin-Lip suspension to disrupt the liposomes, and then the suspension was centrifuged at 12,000 rpm for 15 min. Protein samples were obtained after the lower liquid had been blow-dried with a nitrogen blower and dissolved in 50 μL of deionized water, and zero, standard, and sample wells were set up. Approximately 20 μL of diluent was added to the zero well; 0.025, 0.05, 0.1, 0.2, 0.3, and 0.4 mg/mL protein standard solution was added to the standard well; and 20 μL of the protein sample was added to the sample well. A BCA working solution was added to each well, and the samples were immediately incubated in a water bath at 37 °C for 30 min. The absorbance of each well was measured at 560 nm. The concentration of each protein sample was calculated from the protein standard curve. The Tf grafting rate was calculated as follows:

Grafting Rate (%) = (content of binding Tf/amount of Tf input) × 100%

#### 4.7.3. Encapsulation Efficiency

The encapsulation efficiency of liposomes was determined using a UV–visible spectrophotometer [56]. Here, 1 mL of dialyzed liposomes and 1 mL of non-dialyzed liposomes were added to centrifuge tubes, respectively. Then, 4 mL of methanol was added to disrupt the liposome via ultrasonic treatment. The absorbance of each sample was then measured at 360 nm using a UV-1800 Visible Absorbance Spectrophotometer (Shimadzu, Suzhou, China). A standard Rutin calibration curve was generated by the serial dilution of Rutin standard solution (5, 10, 20, 40, 60, 80, and 100 μg/mL), and the concentration of Rutin in each sample was calculated from the Rutin standard curve. The entrapment efficiency (EE) of Rutin-Lip and Tf-Rutin-Lip was calculated as follows: EE (%) = (amount of Rutin encapsulated within liposomes/Total amount of Rutin) × 100%.

### 4.8. In Vitro Release Studies

The release of Rutin from the nanoliposomes was studied according to the method of Hanafy et al. [56]. Briefly, 5 mL of Rutin, Rutin-Lip, and Tf-Rutin-Lip suspensions were placed in dialysis bags (molecular weight cutoff [MWCO] 8000–14,000 Da) and then immersed in 200 mL of PBS (pH 7.4), which mimics the physiological environment. The solutions were stirred at 100 rpm at 37 °C. At predetermined time intervals (0.5, 1, 2, 4, 6, 8, 10, 24,36, and 48 h), 2 mL of release medium was withdrawn and replaced with the same volume of fresh pH 7.4 PBS to maintain the source sink conditions. The concentration of Rutin in the release medium was determined using a UV–visible spectrophotometer at a wavelength of 360 nm. The percentage of cumulative drug release was measured in triplicate.

*Cumulative percentage* = *Q* (%) = (*Ct Vt*)/(*C*0 *V*0)

where *C*0 represents the initial concentration of Rutin, Rutin-Lip, or Tf-Rutin-Lip in the dialysis bag; *V*0 represents the initial volume of Rutin, Rutin-Lip, or Tf-Rutin-Lip in the dialysis bag; *Ct* represents the concentration of Rutin, Rutin-Lip, or Tf-Rutin-Lip in the release medium at different time points; and *Vt* represents the volume of the release medium.

### 4.9. Cytotoxicity Assay

To assess the effect of Tf-Rutin-Lip, Blank-Lip, Tf-RhB-Lip, and RhB-Lip on the viability of bEnd.3 cells, 1 × 10^4^ bEnd.3 cells/well were seeded into a 96-well plate and incubated for 24 h. Then, 100 μL of different concentrations (125, 25, 5, 1, and 0.2 μg/mL) of liposomes were added, and the mixture was incubated for another 24 h. The cells treated with DMEM served as the control group. After 24 h of incubation, 10% CCK-8 culture solution was added, and the resultant solution was incubated for 30 min (CCK-8, APExBIO, Houston, TX, USA). Finally, the solution was placed into a microplate reader (Thermo, Multiskan SkyHigh, Waltham, MA, USA) to detect the absorbance at 450 nm (OD_450_ nm). The percentage of viable cells was calculated as follows:

$$Cellviabillty\;(\%)=\frac{{OD}_{\mathrm{test}}-{OD}_{\mathrm{blank}}}{{OD}_{\mathrm{control}}-{OD}_{\mathrm{blank}}}\times 100\%$$

where OD_test_ is the absorbance of the cells incubated with nanoliposome suspensions, OD_control_ is the absorbance of the cells incubated with CCK-8 working solution, and OD_blank_ is the absorbance of CCK-8 working solution without bEnd.3 cells.

### 4.10. Cellular Uptake Study

To explore the uptake of the liposomes by bEnd.3 cells in vitro, bEnd.3 cells were seeded in a confocal dish and treated with either a free RhB solution (2 µg/mL) or RhB-Lips at the same RhB concentration and incubated for 4 h at 37 °C (*n* = 3). After being washed twice with cold PBS, the cells were fixed with 4% paraformaldehyde for 15 min. Subsequently, the cells were treated with DAPI solution (2 µg/mL) to label the nucleus and then imaged under a fluorescence microscope. A fluorescence intensity analysis of the acquired images was performed using ImageJ (ImageJ 1.54g).

### 4.11. Transmembrane Transportation Assay

To investigate the influence of liposomes on the integrity and functionality of the blood–brain barrier, the expression of the factors such as *JAM-1*, *ZO-1*, *Claudin-5*, *Occludin*, *MMP-9*, and *MMP-2* [57,58] was detected in bEnd.3 cells treated with RhB-Lip, Tf-RhB-Lip, Blank-Lip, and Tf-Rutin-Lip. In brief, 1.0 × 10^6^ bEnd.3 cells were cultured in 6-well plates. Once the cells had reached confluence, the drug was added and allowed to act for 4 h. Following this, the cells were washed three times with PBS and lysed by adding 1 mL of SparkZol, followed by blowing to disrupt the cells. The lysate was then stored at −80 °C. We used the All-In-One 5× RT MasterMix kit to efficiently reverse-transcribe RNA to cDNA, followed by a precise fluorescent quantitative PCR analysis of the resulting cDNA using the BlasTaq™ 2× qPCR MasterMix kit. The primer information used in this study is listed in Table 8.

An in vitro BBB model was established using bEnd.3 cells and C8-D1A astrocytes to measure the BBB penetration efficiency. In brief, the 1.0 × 10^5^ bEnd.3 cells were cultured in a 12-well Transwell chamber (0.4 µm pore) pretreated with 0.1% gelatin. The integrity of the BBB model was further verified through a 4 h leakage assay and a fluorescein sodium permeability assay. The BBB model was considered successful when the 4 h leakage assay was successful. Following this, three groups (free RhB solution, RhB-Lip and Tf-RhB-Lip; each containing 5 µg/mL) were added to the upper chamber and cultured for 4 h at 37 °C (*n* = 3). The cell in the lower chamber was fixed with 4% paraformaldehyde for 15 min. After fixation, the cells were washed three times with PBS to remove any residual fixative or other impurities. Subsequently, the cells were treated with DAPI solution (2 µg/mL) for a period of time to effectively label the nuclei. Following the DAPI staining, the cells were visualized using an inverted fluorescence microscope (Nikon TS2-FL, Beijing, China). The microscope was used to observe and photograph the fluorescence intensity of the nuclei in the cells within each well [44].

### 4.12. Biodistribution in Mice

To evaluate the in vivo distribution of the Tf-modified liposomes, the liposomes were labeled with near-infrared fluorescent probe ICG. The free ICG, ICG-Lip, and Tf-ICG-Lip (200 µg/mL per mouse) were injected into the mice via the tail vein (*n* = 3). After injection, the mice were anesthetized using chloral hydrate and imaged using the in vivo fluorescence imaging system (IVIS^®^ Lumina III Small Animal Imaging System, manufactured by PerkinElmer, Waltham, MA, USA) at 2, 4, 6, 12, 24, and 36 h post injection [49].

### 4.13. Development and Validation of HPLC Method

To ensure that the HPLC method developed for the analysis of Rutin in rat plasma for pharmacokinetic studies was reliable and reproducible, the method was validated in terms of specificity and selectivity, linearity, precision and accuracy, recovery, and stability.

#### 4.13.1. Chromatographic Conditions

Chromatographic separation was carried out on an Intertsil ODS-3 C18 column (250 mm × 4.6 mm, 4 µm) using a Shimadzu 2050A liquid chromatograph. The analysis was performed with ultraviolet (UV) detection using a diode array detector (DAD) set at 358 nm for Rutin. The mobile phase consisted of methanol and 0.5% H_3_PO_4_ (50:50 *v*/*v*). The flow rate was set at 0.8 mL/min, the injection volume was set at 20 μL, and the column temperature was set at 30 °C. The Rutin retention time was found to be 24.78 min.

#### 4.13.2. Preparation of Standard and Quality Controls

Rutin stock solutions were prepared at a concentration of 1 mg/mL in methanol. A calibration curve was prepared with Rutin standard solutions at concentrations of 0.01, 0.05, 0.1, 0.2, 0.5, 1, 2, and 5 µg/mL. The prepared standard curve was found to be linear, with a correlation coefficient of 0.9998. Quality control (QC) samples were set at low (0.1 µg/mL), medium (0.5 µg/mL), and high (5 µg/mL) concentrations, and were prepared in the same way as the standard concentrations.

#### 4.13.3. Preparation of Plasma Samples

Plasma samples (200 µL each) were extracted using the protein precipitation method with 1.0 mL of methanol. The sample was vortexed for 1 min and then underwent centrifugation for 5 min at 10,000 rpm. The supernatant was transferred into glass tubes and the solvent was removed via evaporation with a gentle stream of air for 2.5 h. The dried residue was reconstituted with 200 µL of the mobile phase by vortexing for 1 min and centrifuging for 5 min at 10,000 rpm. The supernatant was passed through a 0.22 µm organic membrane filter, and a volume of 35 µL was collected and transferred into vials for immediate HPLC analysis.

#### 4.13.4. Specificity and Selectivity

The absence of interference with the matrix and the analyte was ascertained for three sources. The selectivity was evaluated by injecting the blank plasma, plasma spiked with Rutin standard solution, or Rutin standard solution.

#### 4.13.5. Linearity and Sensitivity

The linearity was evaluated by injecting a standard curve with six concentrations of Rutin ranging from 0.05 to 5 µg/mL. The linearity of the curve was generated by using linear least-squares regression (R^2^) analysis. Sensitivity was defined by the lowest concentration of the curve that can be measured and quantified in terms of precision and accuracy. The Rutin concentration of 0.05 µg/mL was determined as the LOD.

#### 4.13.6. Precision and Accuracy

Accuracy and precision were quantitatively represented by the recovery rate and RSD (relative standard deviation), respectively. This study determined the recovery rate and RSD by adding different concentrations of Rutin reference substances to blank plasma samples. Specifically, the addition–recovery test was conducted at three concentration levels: 0.1, 1, and 5 µg/mL. The relative standard deviation (RSD%) was determined for precision and was acceptable for all quality control (QC) samples that did not exceed 15% and 20% for LOQ samples under the acceptance criteria set by FDA guidelines. The accuracy was determined by comparing the calculated concentrations derived from the equation of the calibration curve to the nominal concentration. The acceptance criteria for accuracy were that the mean values of the calculated concentration compared to nominal concentration fell within ±15% for QC samples and ±20% for LOQ.

#### 4.13.7. Stability

To investigate the stability of plasma samples, this study employed repeated freeze–thaw cycles and long-term low-temperature storage (maintained at −80 °C for 30 days). Additionally, rutin standards with concentrations of 0.1, 1, and 5 μg/mL were, respectively, added to blank plasma to further analyze their stability performance under specific conditions.

### 4.14. Pharmacokinetics Study

Healthy SD rats were randomly divided into three groups (*n* = 6). A single dose of Tf-Rutin-Lip, Rutin-Lip, or free Rutin solution (equivalent to 100 mg/kg Rutin) in 200 µL of PBS (pH 7.4) was administered to the mice by gavage (*n* = 6). At prescribed time points post injection (0.5, 1, 2, 4, 6, 12, and 24 h), 0.5 mL of blood samples was collected from the tail vein. The concentration of Rutin in the blood was determined using the HPLC method established above. The data were analyzed using the pharmacokinetic software WinNonlin 8.1.0 (Pharsight Corp., Mountain View, CA, USA), where a non-compartmental model fitting approach was employed to derive the main pharmacokinetic parameters (T_1/2_, T_max_, C_max_, AUC_0-∞_, AUC_0-t_, MRT_0-∞_, and CL) of the two drugs. This approach comprehensively reflected the absorption, distribution, and elimination of the drugs in the rats, providing a comprehensive pharmacokinetic profile for each compound.

### 4.15. Statistical Analysis

The statistical analysis was performed using SPSS 27 software and the data expressed as the mean ± standard deviation (SD). The differences between groups were assessed using a one-way analysis of variance (ANOVA). Post-hoc multiple comparisons were conducted with the Dunnett’s T3. A *p* value < 0.05 was considered to indicate statistical significance.

## 5. Conclusions

In summary, Tf-modified Rutin liposome (Tf-Rutin-Lip) was successfully prepared using the thin-film dispersion method. The Tf-modified liposome effectively penetrated the blood–brain barrier in vivo and in vitro, and it also exhibited good slow-release properties in pharmacokinetic tests. Rutin modified with Tf is expected to enhance brain targeting and may prolong drug circulation time in treating brain disorders. However, the content of Rutin in brain tissue should be measured using HPLC in future studies. Moreover, Tf-Rutin-Lip should be further evaluated in multiple animal models for the treatment of brain diseases.

## Figures and Tables

**Table 1:** Observed responses in Box–Behnken design for Rutin nanolioposomal formulations.

Formula NO.	Independent Variables			Dependent Variables
	A (*w*/*w*)	B (*w*/*w*)	C (°C)	Y (%)
1	0	0	0	68.60
2	−1	−1	0	56.83
3	0	0	0	67.81
4	+1	−1	0	62.28
5	+1	0	−1	65.99
6	0	0	0	71.44
7	0	+1	+1	64.06
8	+1	+1	−1	63.00
9	0	−1	+1	50.37
10	−1	0	−1	52.31
11	0	0	0	72.37
12	0	0	0	69.37
13	+1	0	+1	49.52
14	0	−1	−1	66.78
15	−1	+1	0	57.82
16	−1	0	+1	54.62
17	0	+1	−1	57.12

**Table 2:** Analysis of variance of the fitted linear/quadratic equations for entrapment efficiency (Y) of nanoliposomes.

Source	Sum of Squares	df	Mean Square	F Value	*p*-Value	Prob > F
Model	860.19	9	95.58	35.67	<0.0001	significant
A	46.13	1	46.13	17.21	0.0043	
B	4.12	1	4.12	1.54	0.2550	
C	69.80	1	69.80	26.05	0.0014	
AB	0.0182	1	0.0182	0.0068	0.9366	
AC	88.17	1	88.17	32.90	0.0007	
BC	136.31	1	136.31	50.87	0.0002	
A²	203.61	1	203.61	75.99	<0.0001	
B²	37.43	1	37.43	13.97	0.0073	
C²	227.71	1	227.71	84.98	<0.0001	
Residual	18.76	7	2.68			
Lack of Fit	3.95	3	1.32	0.3554	0.7891	not significant
Pure Error	14.81	4	3.70			
Cor Total	878.95	16				
Adjusted R²	0.9512					
Predicted R²	0.9018					

**Table 3:** Size, PDI, zeta potential, EE, and Tf grafting rate of liposome.

	Blank Liposomes	Rutin-Lip	Tf-Rutin-Lip
Particle Size (nm)	88.8 ± 1.15	112.4 ± 0.30	132.8 ± 1.563
Polydispersity Index (PDI)	0.29 ± 0.044	0.371 ± 0.009	0.398 ± 0.021
Zeta Potential (mV)	0.578 ± 0.234	0.694 ± 0.649	0.787 ± 0.407
EE (%)	*	70.77 ± 0.645	72.68 ± 0.035
Tf Grafting Rat (%)	*	*	54.32 ± 0.067

Note: Values expressed as mean ± SD, *n* = 3; EE: entrapment efficiency. This table uses the “*” symbol uniformly to represent data items that do not exist.

**Table 4:** Recovery and coefficient of variation of rat plasma spiked with Rutin.

Sample	Theoretical Concentration (μg/mL)	Batch Recovery Rate			Intra-Batch Coefficient of Variation (%)	Average Recovery Rate (%)	Inter-Batch Coefficient of Variation (%)
		1	2	3			
plasma	0.1	96.08	97.73	83.40	8.48	91.82	5.57
		92.10	84.60	82.63	5.78		
		100.73	100.27	88.84	6.97		
	1	102.06	101.69	98.08	2.18	101.18	0.51
		98.41	104.61	101.77	3.06		
		97.06	102.13	104.81	3.88		
	5	85.90	89.72	86.08	2.47	92.70	6.60
		90.65	103.71	103.57	7.55		
		91.71	91.56	91.43	0.15		

**Table 5:** Stability data for Rutin (*n* = 3).

	Theoretical Concentration (μg/mL)	Mean Concentration Found (μg/mL)	RSD (%)	Accuracy (%)
Freeze–thaw cycles	0.1	0.0967	1.49	96.76
	1	1.0042	3.90	100.42
	5	4.2922	0.86	85.85
Long-term freezing	0.1	0.0966	5.27	96.67
	1	0.9555	2.82	95.56
	5	4.4929	6.89	89.86

**Table 6:** Pharmacokinetic parameters of Rutin in plasma after administration of different formulations (*n* = 6).

Formulation	Rutin Solution	Rutin-Lip	Tf-Rutin-Lip
T_max_ (h)	2	4	4
C_max_ (µg/mL)	0.134 ± 0.026 ^Aa^	0.210 ± 0.007 ^Bb^	0.267 ± 0.017 ^Cc^
AUC_0-∞_ (µg· h/mL)	1.036 ± 0.269 ^Aa^	2.211 ± 0.367 ^Bb^	2.872 ± 0.458 ^Cc^
AUC_0-t_ (µg· h/mL)	0.901 ± 0.187 ^Aa^	1.885 ± 0.376 ^Bb^	2.324 ± 0.212 ^Bc^
CL (mL/h/kg)	98.94 ± 21.81 ^Bb^	45.21 ± 6.48 ^Aa^	35.63 ± 6.37 ^Aa^
MRT_0-∞_ (h)	6.954 ± 0.822 ^Aa^	7.977 ± 0.544 ^ABb^	8.794 ± 0.630 ^Bb^
T_1/2_ (h)	8.294 ± 2.251	9.037 ± 1.535	9.908 ± 3.286

Peer data labeled with different capital letters indicate highly significant differences between groups (*p* < 0.01), different lowercase letters indicate significant differences (*p* < 0.05), and the same letters or no letters indicate no significant differences (*p* > 0.05).

**Table 7:** Independent variables and their coded levels in the Box–Behnken design.

Independent Variables	Symbols	Coded Levels		
		−1	0	+1
Phospholipid-to-cholesterol (*w*/*w*)	A	2:1	4:1	6:1
Drug-to-lipid ratio (*w*/*w*)	B	1:40	1:50	1:60
Preparation temperature (°C)	C	40	45	50

**Table 8:** Sequence of primers.

Gene	Primer Sequences (5′-3′)	Product Length (bp)	Accession Number
*JAM*-1	F: GTCCTGGTAACACTGATTCTCCTTG	126	NM_172647.2
	R: GGGCTGGCTGTAAATGACCTTC		
*ZO*-1	F: GGCTGTCTCAACTCCTGTAAAA	286	NM_009386.3
	R: TATTCCGACATCATTTCCACCAG		
*Claudin*-5	F: CTGCCTTCCTGGACCACAATA	197	NM_013805.4
	R: GGTAACAAAGAGTGCCACAAGC		
*Occludin*	F: GGCGGCTATGGAGGCTATGG	106	NM_008756.2
	R:CTAAGGAAGCGATGAAGCAGAAGG		
*MMP*-9	F: AGCACGGCAACGGAGAAGG	133	NM_013599.5
	R: TCCTGGTCATAGTTGGCTGTGG		
*MMP*-2	F: CCATGCGGAAGCCAAGATGTG	131	NM_008610.3
	R: GGTTTCAGGGTCCAGGTCAGG		

## Data Availability

All data are available from the corresponding author by request.

## References

[1] Neganova M.E., Aleksandrova Y.R., Sukocheva O.A., Klochkov S.G. (2022). Benefits and limitations of nanomedicine treatment of brain cancers and age-dependent neurodegenerative disorders. Semin. Cancer Biol..

[2] Latos-Brozio M., Masek A. (2019). Structure-Activity relationships analysis of monomeric and polymeric polyphenols (quercetin, rutin and catechin) obtained by various polymerization methods. Chem. Biodivers..

[3] Zhu F. (2016). Chemical composition and health effects of Tartary buckwheat. Food Chem..

[4] An R., Shi C., Tang Y., Cui Z., Li Y., Chen Z., Xiao M., Xu L. (2024). Chitosan/rutin multifunctional hydrogel with tunable adhesion, anti-inflammatory and antibacterial properties for skin wound healing. Carbohydr. Polym..

[5] Tanko Y., Salisu A.I., Mohammed K.A., Musa S.A., Jimoh A., Yusuf R. (2017). Anti-hyperglycaemic effects of rutin on blood glucose, oxidative stress biomarkers and lipid peroxidation in alloxan-induced hyperglycaemic wistar rats. Niger. J. Physiol. Sci..

[6] Ninfali P., Antonelli A., Magnani M., Scarpa E.S. (2020). Antiviral properties of flavonoids and delivery strategies. Nutrients.

[7] Prasad R., Prasad S.B. (2021). Modulatory Effect of rutin on the antitumor activity and genotoxicity of cisplatin in tumor-bearing mice. Adv. Pharm. Bull..

[8] Hu Y., Jia K., Zhou Y., Chen L., Wang F., Yi X., Huang Y., Ge Y., Chen X., Liao D. (2023). Rutin hydrate relieves neuroinflammation in zebrafish models: Involvement of NF-kappaB pathway as a central network. Fish Shellfish Immunol..

[9] Satari A., Ghasemi S., Habtemariam S., Asgharian S., Lorigooini Z. (2021). Rutin: A flavonoid as an effective sensitizer for anticancer therapy; insights into multifaceted mechanisms and applicability for combination therapy. Evid.-Based Complement. Altern. Med..

[10] Ghorbani A. (2017). Mechanisms of antidiabetic effects of flavonoid rutin. Biomed. Pharmacother..

[11] Da S.A., Cerqueira C.P., Das N.O.M., Oliveira J.L., Oliveira A.J., Da S.K., Soares J., Pitanga B., Dos S.S.C., de Faria L.G. (2020). The flavonoid rutin and its aglycone quercetin modulate the microglia inflammatory profile improving antiglioma activity. Brain. Behav. Immun..

[12] Zhao T., He F., Zhao K., Yuxia L., Li H., Liu X., Cen J., Duan S. (2023). A triple-targeted rutin-based self-assembled delivery vector for treating ischemic stroke by vascular normalization and anti-inflammation via ACE2/Ang1-7 signaling. ACS Cent. Sci..

[13] Ola M.S., Ahmed M.M., Ahmad R., Abuohashish H.M., Al-Rejaie S.S., Alhomida A.S. (2015). Neuroprotective effects of rutin in streptozotocin-induced diabetic rat retina. J. Mol. Neurosci..

[14] Santos B.L., Silva A.R., Pitanga B.P., Sousa C.S., Grangeiro M.S., Fragomeni B.O., Coelho P.L., Oliveira M.N., Menezes-Filho N.J., Costa M.F. (2011). Antiproliferative, proapoptotic and morphogenic effects of the flavonoid rutin on human glioblastoma cells. Food Chem..

[15] Santos B.L., Oliveira M.N., Coelho P.L., Pitanga B.P., Da S.A., Adelita T., Silva V.D., Costa M.F., El-Bacha R.S., Tardy M. (2015). Flavonoids suppress human glioblastoma cell growth by inhibiting cell metabolism, migration, and by regulating extracellular matrix proteins and metalloproteinases expression. Chem. Biol. Interact..

[16] Do N.R., Dos S.B., Amparo J., Soares J., Da S.K., Santana M.R., Almeida A., Da S.V., Costa M., Ulrich H. (2022). Neuroimmunomodulatory properties of flavonoids and derivates: A potential action as adjuvants for the treatment of glioblastoma. Pharmaceutics.

[17] Yang Y.F., Xu W., Song W., Ye M., Yang X.W. (2015). Transport of twelve coumarins from angelicae pubescentis radix across a MDCK-pHaMDR cell monolayer-an in vitro model for blood-brain barrier permeability. Molecules.

[18] Park S.N., Lee M.H., Kim S.J., Yu E.R. (2013). Preparation of quercetin and rutin-loaded ceramide liposomes and drug-releasing effect in liposome-in-hydrogel complex system. Biochem. Biophys. Res. Commun..

[19] Sweeney M.D., Sagare A.P., Zlokovic B.V. (2018). Blood-brain barrier breakdown in Alzheimer disease and other neurodegenerative disorders. Nat. Rev. Neurol..

[20] Negahdari R., Bohlouli S., Sharifi S., Maleki D.S., Rahbar S.Y., Khezri K., Jafari S., Ahmadian E., Gorbani J.N., Raeesi S. (2021). Therapeutic benefits of rutin and its nanoformulations. Phytother. Res..

[21] Rakotondrabe T.F., Fan M.X., Muema F.W., Guo M.Q. (2023). Modulating Inflammation-mediated diseases via natural phenolic compounds loaded in nanocarrier systems. Pharmaceutics.

[22] Zou B., Long Y., Gao R., Liu Q., Tian X., Liu B., Zhou Q. (2024). Nanodelivery system of traditional Chinese medicine bioactive compounds: Application in the treatment of prostate cancer. Phytomedicine.

[23] Nisini R., Poerio N., Mariotti S., De Santis F., Fraziano M. (2018). The multirole of liposomes in therapy and prevention of infectious diseases. Front. Immunol..

[24] Ross C., Taylor M., Fullwood N., Allsop D. (2018). Liposome delivery systems for the treatment of Alzheimer’s disease. Int. J. Nanomed..

[25] Guimaraes D., Cavaco-Paulo A., Nogueira E. (2021). Design of liposomes as drug delivery system for therapeutic applications. Int. J. Pharm..

[26] Mathiyazhakan M., Wiraja C., Xu C. (2018). A concise review of gold nanoparticles-based photo-responsive liposomes for controlled drug delivery. Nano-Micro Lett..

[27] Memar M.Y., Dalir A.E., Yekani M., Kouhsoltani M., Sharifi S., Maleki D.S. (2023). Preparation of rutin-loaded mesoporous silica nanoparticles and evaluation of its physicochemical, anticancer, and antibacterial properties. Mol. Biol. Rep..

[28] He R.X., Ye X., Li R., Chen W., Ge T., Huang T.Q., Nie X.J., Chen H.J., Peng D.Y., Chen W.D. (2017). PEGylated niosomes-mediated drug delivery systems for Paeonol: Preparation, pharmacokinetics studies and synergistic anti-tumor effects with 5-FU. J. Liposome Res..

[29] Wicki A., Witzigmann D., Balasubramanian V., Huwyler J. (2015). Nanomedicine in cancer therapy: Challenges, opportunities, and clinical applications. J. Control. Release.

[30] Kobayashi T., Ishida T., Okada Y., Ise S., Harashima H., Kiwada H. (2007). Effect of transferrin receptor-targeted liposomal doxorubicin in P-glycoprotein-mediated drug resistant tumor cells. Int. J. Pharm..

[31] Luo M., Lewik G., Ratcliffe J.C., Choi C., Makila E., Tong W.Y., Voelcker N.H. (2019). Systematic evaluation of transferrin-modified porous silicon nanoparticles for targeted delivery of doxorubicin to glioblastoma. ACS Appl. Mater. Interfaces.

[32] Sawant R.R., Jhaveri A.M., Koshkaryev A., Zhu L., Qureshi F., Torchilin V.P. (2014). Targeted transferrin-modified polymeric micelles: Enhanced efficacy in vitro and in vivo in ovarian carcinoma. Mol. Pharm..

[33] Zhou X., Smith Q.R., Liu X. (2021). Brain penetrating peptides and peptide-drug conjugates to overcome the blood-brain barrier and target CNS diseases. Wiley Interdiscip. Rev.-Nanomed. Nanobiotechnol..

[34] Bermejo-Bescós P., Jiménez-Aliaga K.L., Benedí J., Martín-Aragón S. (2023). A diet containing rutin ameliorates brain intracellular redox homeostasis in a mouse model of Alzheimer’s disease. Int. J. Mol. Sci..

[35] Yuceli S., Yazici G.N., Mammadov R., Suleyman H., Kaya M., Ozdogan S. (2020). The Effect of rutin on experimental traumatic brain injury and edema in rats. In Vivo.

[36] Zhai K., Mazurakova A., Koklesova L., Kubatka P., Busselberg D. (2021). Flavonoids synergistically enhance the anti-glioblastoma effects of chemotherapeutic drugs. Biomolecules.

[37] de Oliveira C., Colenci R., Pacheco C.C., Mariano P.M., Do P.P., Mamprin G., Santana M.G., Gambero A., de Oliveira C.P., Priolli D.G. (2019). Hydrolyzed rutin decreases worsening of anaplasia in glioblastoma relapse. CNS Neurol. Disord.-Drug Targets.

[38] Arowoogun J., Akanni O.O., Adefisan A.O., Owumi S.E., Tijani A.S., Adaramoye O.A. (2021). Rutin ameliorates copper sulfate-induced brain damage via antioxidative and anti-inflammatory activities in rats. J. Biochem. Mol. Toxicol..

[39] Has C., Sunthar P. (2020). A comprehensive review on recent preparation techniques of liposomes. J. Liposome Res..

[40] Sonali, Singh R.P., Singh N., Sharma G., Vijayakumar M.R., Koch B., Singh S., Singh U., Dash D., Pandey B.L. (2016). Transferrin liposomes of docetaxel for brain-targeted cancer applications: Formulation and brain theranostics. Drug Deliv..

[41] Wang Y., Yang Y., Yu Y., Li J., Pan W., Yang X., Zhang Z., Jiang S., Yang X., Wang X. (2020). Transferrin modified dioscin loaded PEGylated liposomes: Characterization and in vitro antitumor effect. J. Nanosci. Nanotechnol..

[42] Craparo E.F., Musumeci T., Bonaccorso A., Pellitteri R., Romeo A., Naletova I., Cucci L.M., Cavallaro G., Satriano C. (2021). MPEG-PLGA nanoparticles labelled with loaded or conjugated rhodamine-B for potential nose-to-brain delivery. Pharmaceutics.

[43] Lochhead J.J., Yang J., Ronaldson P.T., Davis T.P. (2020). Structure, function, and regulation of the blood-brain barrier tight junction in central nervous system disorders. Front. Physiol..

[44] Chen Z.L., Huang M., Wang X.R., Fu J., Han M., Shen Y.Q., Xia Z., Gao J.Q. (2016). Transferrin-modified liposome promotes alpha-mangostin to penetrate the blood-brain barrier. Nanomedicine.

[45] Lopalco A., Cutrignelli A., Denora N., Lopedota A., Franco M., Laquintana V. (2018). Transferrin functionalized liposomes loading dopamine HCl: Development and permeability studies across an in vitro model of human blood–brain barrier. Nanomaterials.

[46] Kazmierczak Z., Szostak-Paluch K., Przybylo M., Langner M., Witkiewicz W., Jedruchniewicz N., Dabrowska K. (2020). Endocytosis in cellular uptake of drug delivery vectors: Molecular aspects in drug development. Bioorg. Med. Chem..

[47] Reinhart M.B., Huntington C.R., Blair L.J., Heniford B.T., Augenstein V.A. (2016). Indocyanine green. Surg. Innov..

[48] Portnoy E., Vakruk N., Bishara A., Shmuel M., Magdassi S., Golenser J., Eyal S. (2016). Indocyanine green liposomes for diagnosis and therapeutic monitoring of cerebral malaria. Theranostics.

[49] Kong L., Li X.T., Ni Y.N., Xiao H.H., Yao Y.J., Wang Y.Y., Ju R.J., Li H.Y., Liu J.J., Fu M. (2020). Transferrin-modified osthole PEGylated liposomes travel the blood-brain barrier and mitigate alzheimer’s disease-related pathology in APP/PS-1 mice. Int. J. Nanomed..

[50] Dos S.R.B., Lakkadwala S., Kanekiyo T., Singh J. (2020). Dual-modified liposome for targeted and enhanced gene delivery into mice brain. J. Pharmacol. Exp. Ther..

[51] Song X.L., Liu S., Jiang Y., Gu L.Y., Xiao Y., Wang X., Cheng L., Li X.T. (2017). Targeting vincristine plus tetrandrine liposomes modified with DSPE-PEG(2000)-transferrin in treatment of brain glioma. Eur. J. Pharm. Sci..

[52] Boonyapiwat B., Sarisuta N., Kunastitchai S. (2011). Characterization and in vitro evaluation of intestinal absorption of liposomes encapsulating zanamivir. Curr. Drug Deliv..

[53] Tang M., Gui Z., Liang X., Yan C., Li X., Li Z., He N., Chang X., Guo J., Gui S. (2021). Pueraria flavones-loaded bile salt liposomes with improved intestinal absorption and oral bioavailability: In vitro and in vivo evaluation. Pharm. Dev. Technol..

[54] Eskandari Z., Bahadori F., Celik B., Onyuksel H. (2020). Targeted nanomedicines for cancer therapy, from basics to clinical trials. J. Pharm. Pharm. Sci..

[55] Zhou L., Shang Y., Wang Y., Wei X. (2024). Transferrin modified PEG–PLGA nanoparticles: Highly effective notoginsenoside R1 formulations for the treatment of ulcerative colitis. J. Pharm Investig..

[56] Hanafy N.A., Sheashaa R.F., Moussa E.A., Mahfouz M.E. (2023). Potential of curcumin and niacin-loaded targeted chitosan coated liposomes to activate autophagy in hepatocellular carcinoma cells: An in vitro evaluation in HePG2 cell line. Int. J. Biol. Macromol..

[57] Zhang Y.M., Xu H., Sun H., Chen S.H., Wang F.M. (2014). Electroacupuncture treatment improves neurological function associated with regulation of tight junction proteins in rats with cerebral ischemia reperfusion injury. Evid.-Based Complement. Altern. Med..

[58] Zhao H.B., Jia L., Yan Q.Q., Deng Q., Wei B. (2020). Effect of clostridium butyricum and butyrate on intestinal barrier functions: Study of a rat model of severe acute pancreatitis with intra-abdominal hypertension. Front. Physiol..

